# Supplementary material for: The genomic origins and evolutionary path to a key innovation in the world’s most venomous snakes
Source: Proc Natl Acad Sci U S A. 2026 Sep 4;123(37):e2621054123. doi: 10.1073/pnas.2621054123 (PMC13578310; doi:10.1073/pnas.2621054123)
Supplement: Supplementary file 1 [file pnas.2621054123.sapp.pdf]

PNAS

Supporting Information for
The genomic origins and evolutionary path to a key innovation in the world's most venomous snakes

Jory van Thiel, Noah L. Dowell, Cara F. Smith, Elda E. Sanchez, and Sean B. Carroll

Correspondence and request for materials should be addressed to Sean B. Carroll
Email: carrolls@hhmi.org.

This PDF file includes:

Supporting text
Figures S1 to S16
Tables S1 to S2
SI References

Supporting text

DNA isolation, library preparation, and DNA sequencing

High-molecular-weight DNA was isolated from *O. microlepidotus* and *B. candidus* blood with the NucleoBond HMW DNA kit (Ref. 740160.20; Macherey-Nagel, Düren, Germany) according to the manufacturer's instructions, and its purity and integrity was validated using gel electrophoresis. Subsequently, library preparation and DNA sequencing were performed by Cold Spring Harbor Laboratory (Laurel Hollow, New York, USA). For *P. textilis*, high-molecular-weight DNA extraction, library preparation, and genome sequencing were commercially performed by the Australian Genome Research Facility (Melbourne, Victoria, Australia). The input DNA for the sequencing libraries was size selected for fragments of ~15 kilo base pairs. The libraries were then sequenced using Pacific Biosciences HiFi technology on a Revio system, with three 25M SMRT Cells for *O. microlepidotus* and *P. textilis*, or two 25M SMRT Cells for *B. candidus*.

RNA isolation, library preparation, and RNA sequencing

Total RNA was isolated from *O. microlepidotus* venom gland and liver tissue using RNeasy Plus Mini Kit (Ref. 74134. QIAGEN, Hilden, Germany) according to the manufacturer's instructions, and its purity and integrity was validated using gel electrophoresis. Each tissue was processed separately to prevent cross-contamination. Library preparation and mRNA sequencing were performed by Cold Spring Harbor Laboratory (Laurel Hollow, New York, USA) using Illumina short-read and PacBio long-read technologies. For short-read Illumina RNA sequencing, two Kapa mRNA libraries were prepared, and each library (representing one tissue) was sequenced (2 x 100 cycles) on a NextSeq 550 system. For Pacific Biosciences Iso-Seq sequencing, two SMRTbell Express kits were used to prepare libraries, and each library (representing one tissue) was sequenced in a PacBio Sequel II cell using 30-hour movie times. Reads were processed using the Pacific Biosciences processing pipeline (https://isoseq.how). The data processing included five steps: consensus generation, demultiplexing, refinement, clustering and collapsing of mapped reads. The genome mapping of clustered reads permitted the identification of sets of gene associated read clusters. Collapsing of many reads into a single consensus sequence occurred only within clustered read sets.

Genome assembly

The *O. microlepidotus* and *P. textilis* genomes were assembled using Canu (version 2.1.1) (1) with the settings “genomeSize=1.8G” for estimated genome size and “pacbio-hifi” for the input reads. The *B. candidus* genome was assembled using HIFIasm (version 0.25.0-r726) (2). Because the *F10* gene region was absent from existing *N. scutatus* assemblies, despite transcript and protein evidence indicating its presence in this species (3-5), we reassembled the genome using an alternative assembler to recover this missing region. To do so, Oxford Nanopore Technologies (ONT) genomic reads (SRR30595366) were retrieved from the Sequence Read Archive (SRA) and assembled with Canu (version 2.3) (6) using the settings “genomeSize=1.8G” and “-nanopore”.

Gene annotations

Homology-based manual gene annotations were performed on both newly generated and publicly available genome assemblies. The barred grass snake (*Natrix helvetica*) assembly (GCF_964273705.1) was used as the non-venomous outgroup species for the *F10*, *F5*, and *PLA2G1B* loci. The newly generated assembly for *N. scutatus* was used exclusively for the *F10* region, whereas an existing assembly (GCA_049901735.1) was used for the other venom gene families. The *F10* gene region was also annotated in several publicly available genomes, including *Aipysurus laevis* (GCA_040207615.1) (7), *Hydrophis major* (GCA_033807585.1) (8), *Hydrophis elegans* (GCA_033807725.1) (8), *Hydrophis curtus* (GCA_037043045.1) (8), and *Hydrophis ornatus* (GCA_037055225.1) (8). Furthermore, the Kunitz-type toxin locus was also annotated in *Charina bottae* (GCA_023362775.1) (9). First, we used the “tblastn” command to identify the contigs, scaffolds, or chromosomes containing candidate venom gene(s) by comparison with reference sequences (10). In these genomic regions, we manually annotated the venom genes and their flanking genes using SnapGene (version 8.0) (GSL Biotech LLC, San Diego, Calfornia, USA). The exons were manually identified through an iterative process of either “blastn” or “tblastn”, with reference protein or transcript sequences as queries, and were later refined by identifying exon–intron boundaries. Because of the error-prone nature of the *N. scutatus* ONT reads, small exonic indels that disrupted an otherwise intact reading frames were manually corrected. Most of these indels occurred in homopolymeric regions and were therefore considered to be technical artifacts rather than true biological variation. Finally, we obtained the coding DNA sequences (CDSs), corresponding to the mRNA coding regions, and their

predicted protein translations using the “Splice to Remove Introns…” function implemented in SnapGene.

Transcriptomics

To qualitatively assess whether the B-domains of both *F5* paralogs are alternatively spliced, we analyzed full-length RNA transcripts in both venom gland and liver tissues of *O. microlepidotus*. From this curated Isoseq dataset, all *F5* transcripts were identified using the “blastn” command with the *F5* paralogs from *O. microlepidotus* genome as the query sequences (10). The identified transcripts were extracted, aligned with *F5* gene annotations in AliView (version 1.30) (11), and subsequently manually analyzed for variation in exon 13 (which encodes the entire B-domain).

To quantify relative expression levels of *F10*, *F7* and *F5* genes and respective isoforms, we counted full-length RNA reads in the venom gland and liver tissues of *O. microlepidotus*. We used full-length RNA transcript counts as a semi-quantitative proxy for their gene expression, which allowed us to unambiguously identify and discriminate between highly similar paralogs and isoforms. All transcripts corresponding to each gene were identified using the “blastn” command (10), extracted, aligned with annotated CDSs in AliView (11), and quantified by the same software, with manual verification of transcript identity to prevent any misassignments. To validate this approach, we used several known liver-expressed genes (*F2*, *F9*, *PROZ* and *ALB*) as controls.

To verify venom gland expression of annotated venom toxin genes other than *F10*, *F5*, and *F7*, short RNA reads were mapped to the annotated CDSs. RNA-seq data from *O. microlepidotus* were generated in this study, whereas equivalent data for adult *P. textilis* were retrieved from the SRA database (accessions SRR33084406 and SRR33084395) (12) . First, low-quality reads and adapter sequences were removed using Trimmomatic (version 0.40-rc1) (13) with the following settings: “ILLUMINACLIP:TruSeq3-PE-2.fa:2:30:10”, “LEADING:5”, “TRAILING:5”, “SLIDINGWINDOW:4:30”, “MINLEN:40”. Next, we evaluated read quality and confirmed that adapters had been correctly removed using FastQC (version 0.12.1). We then prepared transcript reference sequences from CDSs of all annotated genes using RSEM (version v1.3.1) (14). Subsequently, processed RNA-seq reads were mapped using RSEM with Bowtie2 (version 2.4.4) (15) with the sensitivity level set to “very sensitive” for the CDS datasets to estimate relative gene expression levels in venom gland and liver tissues of *O. microlepidotus* and *P. textilis.*

To search for additional evidence of *F10* and *F5* expression in *P. textilis* venom gland tissue, we mapped short RNA-seq reads to the corresponding genomic loci. The processed RNA-seq reads were aligned to all contigs in our *P. textilis* genome assembly using the BWA-MEM algorithm (16). Using the Samtools "view" command (17), paired-end reads with a mapping quality (MAPQ) score greater than 20 were retained and sorted, and only the reads mapping to the two contigs containing the *F10* and *F5* genes were extracted. Mapped reads were subsequently visualized and inspected using Integrative Genomics Viewer (IGV; version 2.16.0) (18, 19) to search for reads mapping to regions unique to the *F10* or *F5* genes and not to their respective paralogs.

Proteomics

In order to search for evidence of the hemostatic clotting factor proteins (Factor X, Factor V, and Factor VII) in venoms, we reanalyzed publicly available proteomic data from seven *P. textilis* venom samples (12). Raw mass spectrometry data from trypsin-digested venom protein peptides were retrieved from the PRoteomics IDEntifications (PRIDE) database (dataset identifier PXD063001). Fragmentation spectra were searched against hypothetical protein translations derived from our *P. textilis* gene annotations using the MSFragger-based FragPipe computational platform (20, 21).

Phylogenetic analyses

Bayesian phylogenetic analyses were performed for factor X, group I PLA_2, and Kunitz-type toxins. Hypothetical protein translations from our genome annotations were aligned using the Multiple Sequence Comparison by Log-Expectation (MUSCLE) algorithm (22) in AliView (version 1.30) (11). Phylogenetic analyses were conducted in MrBayes (version 3.2.7a) (23). Signal peptides of group I PLA_2 sequences were manually removed from the alignments. Amino-acid substitution models were sampled using Bayesian model averaging (aamodelpr = mixed). The Markov chain Monte Carlo algorithm was run for 15 million generations, sampling every 100 generations, with four independent runs of four chains each (three hot, one cold), a heating temperature of 0.2, and a burn-in of 25%. All analyses reached convergence, as indicated by meeting the convergence threshold (stopval = 0.01). The consensus phylogenies were constructed using contype=halfcompat (for factor X and group I PLA_2) and contype=allcompat (for Kunitz-type toxins).

Figures

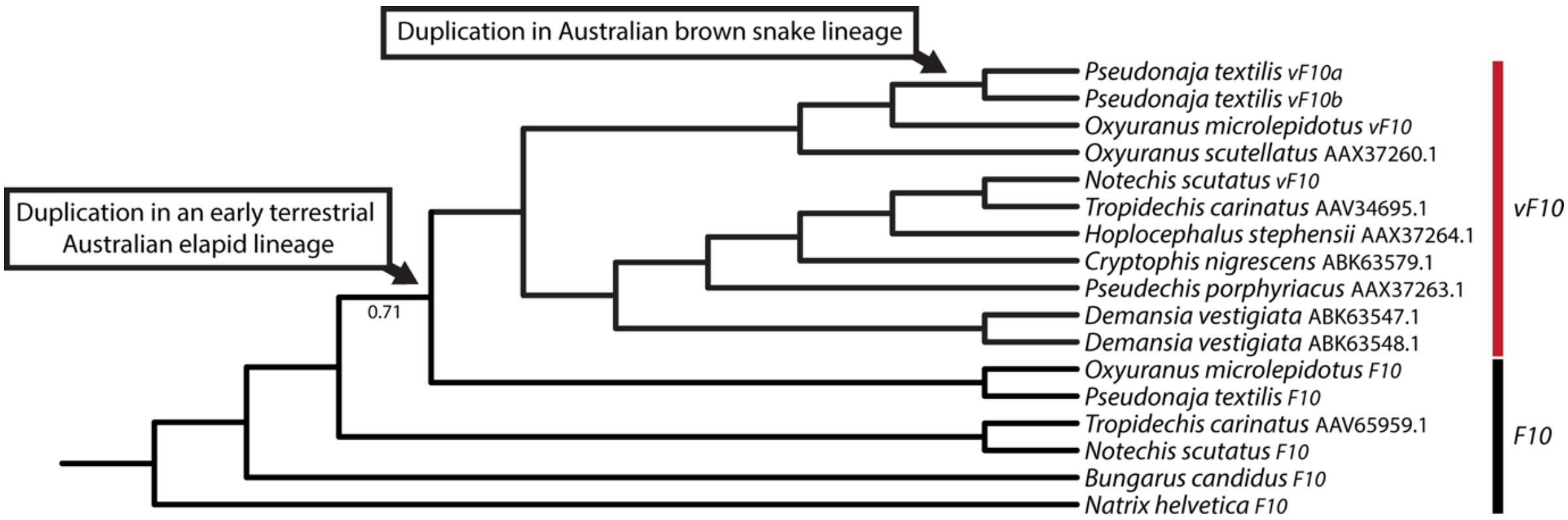

Fig. S1. A venom-expressed *F10* gene was duplicated in the brown snake lineage (*Pseudonaja* spp.). A protein phylogeny based on hypothetical translations of the *F10* genes and full-length cDNA sequences from distinct Australian elapid species. At the base of the phylogeny, there is a cluster of *F10* genes (encoding the hemostatic protein), and nested within it, a well-supported monophyletic clade consisting of *vF10* (encoding the venom toxin). In the venom gene clade, the *vF10b* gene clusters with *vF10a*, indicating that *vF10b* arose through duplication of the venom-expressed *vF10a* and not from the liver-expressed *F10* gene, a scenario that has previously been proposed (24). The phylogeny was rooted with *N. helvetica F10* protein sequence as the outgroup. Only Bayesian posterior probabilities below 1.00 are shown.

	1 76
Pseudonaja textilis vF10a	MAPQLLLCLILTFLWSLPEAESNVFLKSKVANRFLQRTKR**ANSLVEEFKSGNIERECIEERCSKEEAREVFEDDEK**
Pseudonaja textilis vF10b	MAPQLLLCLILTFLWSLPEAESNVFLKSKVANRFLQRTKR**ANSLVEEFKSGNIERECIEERCSKEEAREVFEDDEK**
Oxyuranus microlepidotus vF10	MAPQLLLCLILTFLWSLPEAESNVFLKSKVANRFLQRTKR**ANSLFEEFRSGNIERECIEERCSKEEAREVFEDDEK**
Notechis scutatus vF10	MAPQLLLCLILTFLWSLPEAESNVFLKSKVANRFLQRTKR**SNSLFEEIRPGNIERECIEEKCSKEEAREVFEDNEK**
Pseudonaja textilis F10	MAPQLLLCLILTFLWSLPEAESNVFLKSKVANRFLQRTKR**ANSLFEEFKPGNIERECIEERCSKEEAREAFEDDEK**
Oxyuranus microlepidotus F10	MAPQLLLCLILTFLWSLPEAESNVFLKSKVANRFLQRTKR**ANSLFEEFKPGNIERECIEERCSKEEAREAFEDDEK**
Notechis scutatus F10	MAPQLLLCLILTFLWSLPEAESNVFLKSKVANRFLQRTKR**ANSLFEEFKAGNIERECIEERCSKEEAREAFEDNEK**
Aipysurus laevis F10	MAPQLLLCLILTFLWSLPEAESNVFLKSKVANRFLQRTKR**ANSLFEEFKAGNIERECIEERCSKEEAREAFEDNEK**
Hydrophis major F10	MAPQLLLCLIFTFLWSLPEAESNVFLKSKVANRFLQRTKR**ANSLFEEFKAGNIERECIEERCSKEEAREAFEDNEK**
Hydrophis elegans F10	MAPQLLLCLIFTFLWSLPEAESNVFLKSKVANRFLQRTKR**ANSLFEEFKAGNIERECIEERCSKEEAREAFEDNEK**
Hydrophis curtus F10	MAPQLLLCLIFTFLWSLPEAESNVFLKSKVANRFLQRTKR**ANSLFEEFKAGNIERECIEERCSKEEAREAFEDNEK**
Hydrophis ornatus F10	MAPQLLLCLIFTFLWSLPEAESNVFLKSKVANRFLQRTKR**ANSLFEEFKAGNIERECIEERCSKEEAREAFEDNEK**
Bungarus candidus F10	MAPQLLLCLILTFLWSLPEAESNVFLKSKVANRFLQRTKR**ANSLFEEIKPGNIERECREERCSKEEAREAFENNEE**
Natrix helvetica F10	MAPQLLLCLILTFLWSLPNAESNVFLKGKVANRFLQRTKR**ANSIFEEFKAGNIERECIEERCSKEEAREAFEDQEK**

	77 152
Pseudonaja textilis vF10a	TETFWNVYVDGDQCSSNPCHYRGICKDGIGSYTCTCLSGYEGKNCERVLYKSCRVDNGNCWHFCKSVQNDIQCSCA
Pseudonaja textilis vF10b	TETFWNVYVDGDQCSSNPCHYRGICKDGIGSYTCTCLSGYEGKNCERVLYKSCRVDNGNCWHFCKHVQNDIQCSCA
Oxyuranus microlepidotus vF10	TETFWNVYVDGDQCSSNPCHYRGTCKDGIGSYTCTCLFGYEGKNCERVLYKSCRVDNGNCWHFCKPVQNDIQCSCA
Notechis scutatus vF10	TETFWNVYVDGDQCSSNPCHYRGTCKDGIGSYTCTCLPNYEGKNCEKVLYQSCRVDNGNCWHFCKRVQSETQCSCA
Pseudonaja textilis F10	TETFWNVYVDGDQCSSNPCHYGGTCKDGIGSYTCTCLSGYEGKNCEYVLYKSCRVDNGDCWHFCKPVQNGIQCSCA
Oxyuranus microlepidotus F10	TETFWNVYVDGDQCSSNPCHYGGTCKDGIGSYTCTCLSGYEGKNCEYVLYKSCRVDNGNCWHFCKPVQNGIQCSCA
Notechis scutatus F10	TETFWNVYVDGDQCSSNPCHYGGTCKDGIGSYTCTCLAGYEGKNCQYVLYQSCRVDNGNCWHFCKRVQSEIQCSCA
Aipysurus laevis F10	TETFWNVYVDGDQCSSNPCHYGGTCKDGIGSYTCTCLAGYEGKNCQYVLYKSCRVDNGNCWHFCKPVQNEIQCSCA
Hydrophis major F10	TETFWNVYVDGDQCSSNPCHYGGTCKDGIGSYTCTCLAGYEGKNCQYVLYKSCRVDNGNCWHFCKPVQNEIQCSCA
Hydrophis elegans F10	TETFWNVYVDGDQCSSNPCHYGGTCKDGIGSYTCTCLAGYEGKNCQYVLYKSCRVDNGNCWHFCKPVQNEIQCSCA
Hydrophis curtus F10	TETFWNVYVDGDQCSSNPCHYGGTCKDGIGSYTCTCLAGYEGKNCQYVLYKSCRVDNGNCWHFCKPVQNEIQCSCA
Hydrophis ornatus F10	TETFWNVYVDGDQCSSNPCHYGGTCKDGIGSYTCTCLAGYEGKNCQYVLYKSCRVDNGNCWHFCKPVQNEIQCSCA
Bungarus candidus F10	TEKFWNIYVDGDQCSSNPCHYGGTCKDGIGSYTCTCLAGYQGKNCEYIIYKSCRVDNGNCWHFCKLVQSEIQCSCA
Natrix helvetica F10	TENFWNVYVDGDQCLSNPCHYGATCKDGIGNFSCTCLAGYQGKTCESFILRSCRLDNGNCWHFCKSDQNKIHCSCA

	153 228
Pseudonaja textilis vF10a	EGYLLGEDGHSCVAGGNFSCGRNIKTRNKR**EASLPDF-----------------------------VQSHNATLLK**
Pseudonaja textilis vF10b	EGYLLGEDGHSCVAGGNFSCGRNIKTRNKR**EANLPDF-----------------------------VQSQNATLLK**
Oxyuranus microlepidotus vF10	EGYLLGEDGHSCVAGGNFSCGRNIKTRNKR**EASLPDF-----------------------------VQSQNATLLK**
Notechis scutatus vF10	ESYRLGVDGHSCVAEGDFSCGRNIKARNKR**EASLPDF-----------------------------VQSQKATLLK**
Pseudonaja textilis F10	ESYLLGEDGHSCVAGGDFSCGRNIKTRNKR**EANLPDFQTDFSDDYDEIDENNFVETPTNFSGLVLTVQSQNATLLK**
Oxyuranus microlepidotus F10	ESYLLGEDGHSCVAGGDFSCGRNIKTRNKR**EANLPDFQTDFSDDYDEIDENNFVETPTNFSGLVPTVQSQNATLLK**
Notechis scutatus F10	ESYLLGDDGHSCVAGGDFSCGRNIKARNKR**EASLPDFQTDFSDDYDAIDENNFVETPTNFSGLVPTVQSQNATLLK**
Aipysurus laevis F10	ESYLLGDDGYSCVAEDDFSCGRNIKARNKR**EASLPDFQTDFSDDYDAIDENNFVETPTNFSGLVPTVQSQNASLLK**
Hydrophis major F10	ESYLLGDDGYSCVAEDDFSCGKNIKARNKR**EASLPDFQTDFSDDYDAIDENNFVETPTNFSGLVPTVQSQNANLLK**
Hydrophis elegans F10	ESYLLGDDGYSCVAEDDFSCGKNIKARNKR**EASLPDFQTDFSDDYDAIDENNFVETPTNFSGLVPTVQSQNANLLK**
Hydrophis curtus F10	ESYLLGDDGYSCVAEDDFSCGKNIKARNKR**EASLPDFQTDFSDDYDAIDENNFVETPTNFSGLVPTVQSQNANLLK**
Hydrophis ornatus F10	ESYLLGDDGYSCVAEDDFSCGKNIKARNKR**EASLPDFQTDFSDDYDAIDENNFVETPTNFSGLVPTVQSQNANLLK**
Bungarus candidus F10	ESYVLGDDEYSCVAEDDFSCGRNIKARNKR**EASLPDFQTEFSEDYDAIDENNFVETSTNFSSLVPTLQSRNATSLK**
Natrix helvetica F10	EGYILGDDGQSCVAGGDFSCGRNIKARNKR**EASLVDFQEDFSDDYDVIEEESFVETPTNFSSLVPIMQHRNATKPR**

	229 304
Pseudonaja textilis vF10a	**KSDNPSPDIR**IVNGMDCKLGECPWQAALVDDKKGVFCGGTILSPIYVLTAAHCINETETISVVV-GEIDRSRAETG
Pseudonaja textilis vF10b	**KSDNPSPDIR**IVNGMDCKLGECPWQAALVDEKEGVFCGGTILSPIYVLTAAHCINETETISVVV-GEIDKSRIETG
Oxyuranus microlepidotus vF10	**KSDNPSPDIR**IVNGMDCKLGECPWQAVLVDEKEGVFCGGTILSPIYVLTAAHCINQTEKISVVV-GEIDKSRVETG
Notechis scutatus vF10	**KSDNPSPDIR**IVNGMDCKLGECPWQAVLINEKGEVFCGGTILSPIHVLTAAHCINQTKSVSVIV-GEIDISRKETR
Pseudonaja textilis F10	**KSDNPSPDIR**VVNGTDCKLGECPWQALLLNDEGDGFCGGTILSPIYVLTAAHCINQTKYITVVV-GEIDISSKKTG
Oxyuranus microlepidotus F10	**KSDNPSPDIR**VVNGTDCKLGECPWQALLINDQGDGFCGGTILSPIYVLTAAHCINQTKYIRVVV-GEIDISRKKTG
Notechis scutatus F10	**KSDNPSPDIR**VVNGTDCKLGECPWQALLINDQGDGFCGGTILSPIYVLTAAHCINQTKYIRVVV-EEIDISRKETR
Aipysurus laevis F10	**KSDNPSPDIR**VVNGTDCKLGECPWQALLINDQGDGFCGGTILSPIYVLTAAHCINQTKYIRVVV-GEIDISRKKTG
Hydrophis major F10	**KSDNPSPDVR**VVNGTDCKQGECPWQVLLINDRGDGFCGGTILSPIYVLTAAHCINQTKYIRVVVAGEIDISRKKTG
Hydrophis elegans F10	**KSDNPSPDIR**VVNGTDCKQGECPWQALLINDRGDGFCGGTILSPIYVLTAAHCINQTKYIRVVV-GEIDISRKKTG
Hydrophis curtus F10	**KSDNPSPDIR**VVNGTDCKQGECPWQALLINDRGDGFCGGTILSPIYVLTAAHCINQTKYIRVVV-GEIDISRKKTG
Hydrophis ornatus F10	**KSDNPSPDIR**VVNGTDCKQGECPWQALLINDRGDGFCGGTILSPIYVLTAAHCINQTKYIRVVV-GEIDISRKKTG
Bungarus candidus F10	**KSNNPSPEIR**VVNGTDCKLGECPWQALLLNDQGDGFCGGTILSSIYVLTAAHCINQTKHITVVV-GEVDISSKRNG
Natrix helvetica F10	**KSDNSNPEGR**VVNGTDCKPGECPWQALLINDQGDGFCGGTVLSPMYVLTAAHCINQTTRIKVVV-GEVDISSKKTG

```
                                305                                                                        380
Pseudonaja textilis vF10a       PLLSVDKVYVHKKFVPPKKSQEFYEKFDLVSYDYDIAIIQMKTPIQFSENVVPACLPTADFANQVLMKQDFGIVSG
Pseudonaja textilis vF10b       PLLSVDKIYVHKKFVPPQKAY----KFDLAAYDYDIAIIQMKTPIQFSENVVPACLPTADFANQVLMKQDFGIVSG
Oxyuranus microlepidotus vF10   HLLSVDKIYVHKKFVPPKKGYKFYEKFDLVSYDYDIAIIQMKTPIQFSENVVPACLPTADFANQVLMKQDFGIISG
Notechis scutatus vF10          RLLSVDKIYVHTKFVPPNYYY-VHQNFDRVAYDYDIAIIRMKTPIQFSENVVPACLPTADFANEVLMKQDSGIVSG
Pseudonaja textilis F10         RLHSVDKIYVHQKFVP-------------ATYDYDIAIIQLKTPIQFSENVVPACLPTADFANQVLMKQNFGIVSG
Oxyuranus microlepidotus F10    RLLSVDKIYVHQKFVP-------------ATYDYDIAIIQLKTPIQFSENVVPACLPTADFANQVLMKQNFGIVSG
Notechis scutatus F10           RLLSVDKIYVHQKFVP-------------STYDYDIALIQMKTPIQFSENVVPACLPTADFANQVLMKQDFGIVSG
Aipysurus laevis F10            PL-SVDKIYVHKNFVP-------------TTYDYDIALIQLKTAIQFSENVVPACLPTADFANQVLMKQDFGIVSG
Hydrophis major F10             -LLSVDKIYVHKNFVP-------------TTYDYDIALIQLKTAIQFSENVVPACLPTADFANQVLMKQDFGIVSG
Hydrophis elegans F10           -LLSVDKIYVHKNFVP-------------TTYDYDIALIQLKTAIQFSENVVPACLPTADFANQVLMKQDFGIVSG
Hydrophis curtus F10            -LLSVDKIYVHKNFVP-------------TTYDYDIALIQLKTAIQFSENVVPACLPTADFANQVLMKQDFGIVSG
Hydrophis ornatus F10           -LLSVDKIYVHKNFVP-------------TTYDYDIALIQLKTAIQFSENVVPACLPTADFANQVLMKQDFGIVSG
Bungarus candidus F10           RLIAVDKIYVHQKFVP-------------ATYDYDIAIIQLKTPIQFSENVVPACLPTADFANQVLMKQNFGIVSG
Natrix helvetica F10            HLLAVNKIYVHQKFVL-------------ATYDYDIAIIQLKTPIRFSENVIPACLPTADFANQVLMKQDSGVISG

                                381                                                                        456
Pseudonaja textilis vF10a       FGGIFERGPNSKTLKVLKVPYVDRHTCMLSSNFPITPTMFCAGYDTLPQDACQGDSGGPHITAYRDTHFITGIVSW
Pseudonaja textilis vF10b       FGRIFEKGPKSKTLKVLKVPYVDRHTCMVSSETPITPNMFCAGYDTLPRDACQGDSGGPHTTVYRDTHFITGIVSS
Oxyuranus microlepidotus vF10   FGRIFEKGPKSNTLKVLKVPYVDRHTCMVSSESPITPTMFCAGYDTLPRDACQGDSGGPHITAYRDTHFITGIVSW
Notechis scutatus vF10          FGRIRFKEPTSNTLKVITVPYVDRHTCMLSSDFRITQNMFCAGYDTLPQDACQGDSGGPHITAYRDTHFITGIISW
Pseudonaja textilis F10         FGRTRERGKTSNTLKVVTLPYVDRHTCMLSSNFPITQNMFCAGYDTLPQDACQGDSGGPHITAYRDTHFITGIVSW
Oxyuranus microlepidotus F10    FGRTRERGQTSNTLKVVTLPYVDRHTCMLSSNFPITQNMFCAGYDTLPQDACQGDSGGPHITAYRDTHFITGIVSW
Notechis scutatus F10           FGRIRERGQTSNTLKVITLPYVDRHTCMLSSNFPITQNMFCAGYNTLPQDACQGDSGGPHITAYRDTHFITGIISW
Aipysurus laevis F10            FGRTRERGQTSKTLKVVTLPYVDRHTCMLSSNFPITQNMFCAGYNTLPQDACQGDSGGPHITAYRDTHFITGIISW
Hydrophis major F10             FGRTRERGKTSNTLKVVTLPYVDRHTCMLSSNFPITQNMFCAGYNTLPQDACQGDSGGPHITAYRDTHFITGIISW
Hydrophis elegans F10           FGRTRERGKTSNTLKVVTLPYVDRHTCMLSSNFPITQNMFCAGYNTLPQDACQGDSGGPHITAYRDTHFITGIISW
Hydrophis curtus F10            FGRTRERGKTSNTLKVVTLPYVDRHTCMLSSNFPITQNMFCAGYNTLPQDACQGDSGGPHITAYRDTHFITGIISW
Hydrophis ornatus F10           FGRTRERGKTSNTLKVVTLPYVDRHTCMLSSNFPITQNMFCAGYNTLPQDACQGDSGGPHITAYRDTHFITGIISW
Bungarus candidus F10           FGRTRERGRTSNTLKVVTLPYVDRHICKISSNFPITENMFCAGYNTLPQDACQGDSGGPHITAYRDTHFITGIISW
Natrix helvetica F10            FGRIKERGRTSNTLKMVTLPYVDRHVCKLSSNFPITENMFCAGYRTLAQDACQGDSGGPHVTAYSDTYFVTGIISW

                                457                                     497
Pseudonaja textilis vF10a       GEGCARKGRYGIYTKLSKFIPWIKRIMRQKLPSTESSTGRL
Pseudonaja textilis vF10b       GEGCARNGKYGIYTKLSKFIPWIKRIMRQKLPSTESSTGRL
Oxyuranus microlepidotus vF10   GEGCAKKGKYGIYTKVSKFILWIKRIMRQKLPSTESSTGRL
Notechis scutatus vF10          GEGCARKGKYGVYTKVSRFIPWIKKIMSLK-----------
Pseudonaja textilis F10         GEGCAQTGKYGVYTKVSKFILWIKRIIRQKQPSTESSTGQL
Oxyuranus microlepidotus F10    GEGCAQTGKYGVYTKVSKFILWIKRIMRQKLPSTESSTGRL
Notechis scutatus F10           GEGCAQTGKYGVYTKVSRFILWIKRIMRLKLPSTESSTGRL
Aipysurus laevis F10            GEGCAQTGKYGVYTKVSRFILWIKRIMRLKIPSTE------
Hydrophis major F10             GEGCAQTGKYGVYTKVSRFILWIKRIMRLKIPSTESSTGRL
Hydrophis elegans F10           GEGCAQTGKYGVYTKVSRFILWIKRIMRLKIPSTESSTGRL
Hydrophis curtus F10            GEGCAQTGKYGVYTKVSRFILWIKRIMRLKTPSTESSTGRL
Hydrophis ornatus F10           GEGCAQTGKYGVYTKVSRFILWIKRIMRLKIPSTESSTGRL
Bungarus candidus F10           GEGCAQPDKYGVYTKVSKFIPWIKRIMRQKLPGTDSSSGRL
Natrix helvetica F10            GEGCAQKDKYGVYTKVSKFIPWIKRIMRQKVPRTESGTARP
```

Fig. S2. The venom factor X proteins have a truncated activation peptide region. Amino acid sequence alignment of hypothetical protein translations of *F10* genes**.** Residues shown in grey encode the signal peptide and propeptide regions, and residues in bold indicate the activation peptide region, which is intact in proteins encoded by the *F10* genes but exhibits a 29-amino-acid deletion in proteins encoded by *vF10* genes.

Oxyuranus microlepidotus vF10	MAPQLLLCLILTFLWSLPEAESNVFLKSKVANRFLQRTKRANSLFEEF**RS**GNIERECIEERCSKEEARE**V**FEDDEK
Oxyuranus microlepidotus F10	MAPQLLLCLILTFLWSLPEAESNVFLKSKVANRFLQRTKRANSLFEEF**KP**GNIERECIEERCSKEEARE**A**FEDDEK
transcript/802606	MAPQLLLCLILTFLWSLPEAESNVFLKSKVANRFLQRTKRANSLFEEF**KP**GNIERECIEERCSKEEARE**A**FEDDEK
Oxyuranus microlepidotus vF10	TETFWNVYVDGDQCSSNPCHY**R**GTCKDGIGSYTCTCL**F**GYEGKNCE**R**VLYKSCRVDNGNCWHFCKPVQN**D**IQCSCA
Oxyuranus microlepidotus F10	TETFWNVYVDGDQCSSNPCHY**G**GTCKDGIGSYTCTCL**S**GYEGKNCE**Y**VLYKSCRVDNGNCWHFCKPVQN**G**IQCSCA
transcript/802606	TETFWNVYVDGDQCSSNPCHY**G**GTCKDGIGSYTCTCL**S**GYEGKNCE**Y**VLYKSCRVDNGNCWHFCKPVQN**G**IQCSCA
Oxyuranus microlepidotus vF10	E**G**YLLGEDGHSCVAGG**N**FSCGRNIKTRNKREA**S**LPDF-----------------------------VQSQNATLLK
Oxyuranus microlepidotus F10	E**S**YLLGEDGHSCVAGG**D**FSCGRNIKTRNKREA**N**LPDF**QTDFSDDYDEIDENNFVETPTNFSGLVPT**VQSQNATLLK
transcript/802606	E**S**YLLGEDGHSCVAGG**D**FSCGRNIKTRNKREA**N**LPDF**QTDFSDDYDEIDENNFVETPTNFSGLVPT**VQSQNATLLK
Oxyuranus microlepidotus vF10	KSDNPSPDIR**I**VNG**M**DCKLGECPWQA**VLVDEKEGV**FCGGTILSPIYVLTAAHCINQT**EK**I**S**VVVGEID**K**SR**VE**TG**H**
Oxyuranus microlepidotus F10	KSDNPSPDIR**V**VNG**T**DCKLGECPWQA**LLINDQGDG**FCGGTILSPIYVLTAAHCINQT**KY**I**R**VVVGEID**I**SR**KK**TG**R**
transcript/802606	KSDNPSPDIR**V**VNG**T**DCKLGECPWQA**LLINDQGDG**FCGGTILSPIYVLTAAHCINQT**KY**I**R**VVVGEID**I**SR**KK**TG**R**
Oxyuranus microlepidotus vF10	LLSVDKIYVH**K**KFVP**PKKGYKFYEKFDLVS**YDYDIAIIQ**M**KTPIQFSENVVPACLPTADFANQVLMKQ**D**FGI**I**SGF
Oxyuranus microlepidotus F10	LLSVDKIYVH**Q**KFVP-------------**AT**YDYDIAIIQ**L**KTPIQFSENVVPACLPTADFANQVLMKQ**N**FGI**V**SGF
transcript/802606	LLSVDKIYVH**Q**KFVP-------------**AT**YDYDIAIIQ**L**KTPIQFSENVVPACLPTADFANQVLMKQ**N**FGI**V**SGF
Oxyuranus microlepidotus vF10	GR**IF**E**K**G**PK**SNTLKV**LKV**PYVDRHTCM**V**SS**ES**PIT**PT**MFCAGYDTLP**R**DACQGDSGGPHITAYRDTHFITGIVSWG
Oxyuranus microlepidotus F10	GR**TR**E**R**G**QT**SNTLKV**VTL**PYVDRHTCM**L**SS**NF**PIT**QN**MFCAGYDTLP**Q**DACQGDSGGPHITAYRDTHFITGIVSWG
transcript/802606	GR**TR**E**R**G**QT**SNTLKV**VTL**PYVDRHTCM**L**SS**NF**PIT**QN**MFCAGYDTLP**Q**DACQGDSGGPHITAYRDTHFITGIVSWG
Oxyuranus microlepidotus vF10	EGCA**KK**GKYG**I**YTKVSKFILWIKRIMRQKLPSTESSTGRL
Oxyuranus microlepidotus F10	EGCA**QT**GKYG**V**YTKVSKFILWIKRIMRQKLPSTESSTGRL
transcript/802606	EGCA**QT**GKYG**V**YTKVSKFILWIKRIMRQKLPSTESSTGRL

Fig. S3. The hemostatic *F10* gene is transcribed in inland taipan (*Oxyuranus microlepidotus*) venom gland tissue. Protein sequence alignment comparing the hypothetical protein translations of the *F10* and *vF10* paralogs of *O. microlepidotus* with a representative full-length translated Iso-seq read corresponding to hemostatic factor X from venom gland tissue. The alignment provides qualitative evidence that full-length *F10* transcripts are expressed in venom gland tissue. Amino acid residues that differ between the *F10* and *vF10* sequences are shown in bold.

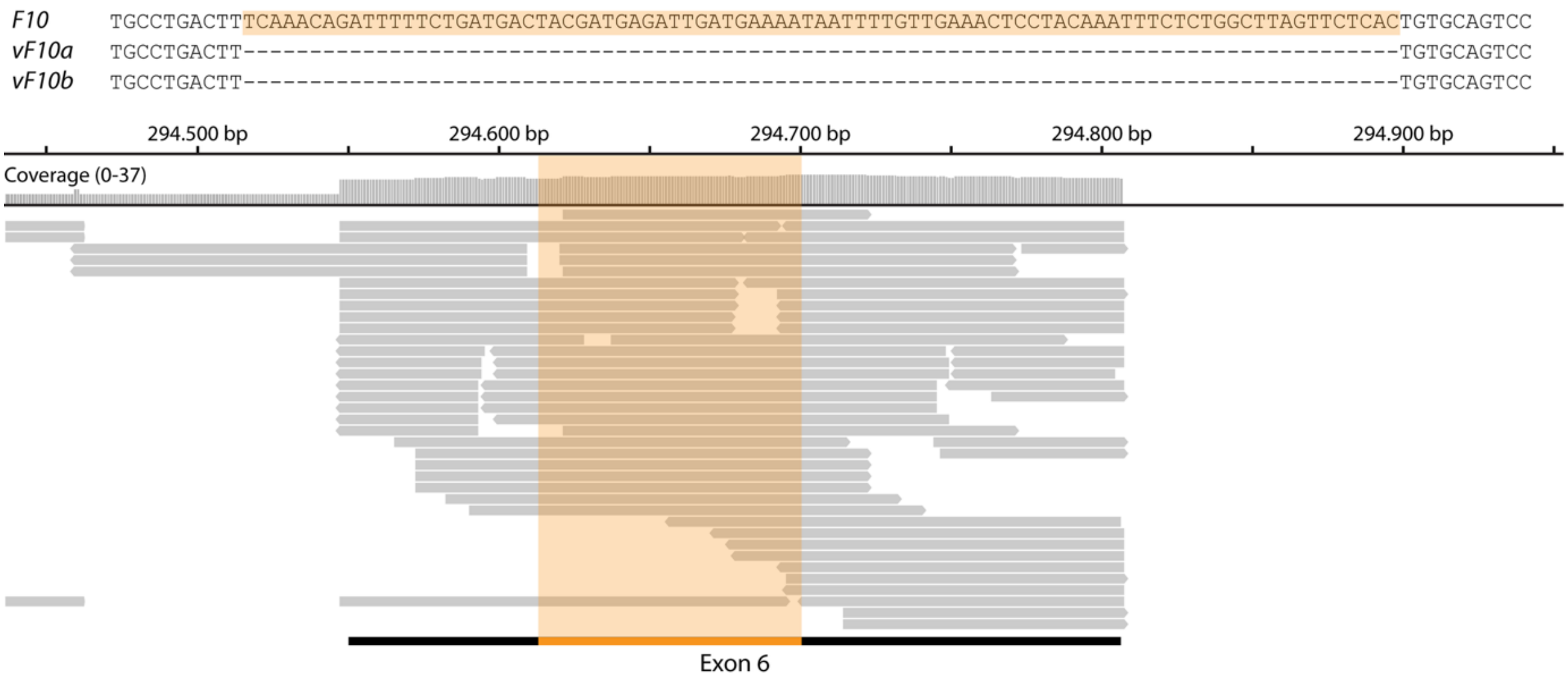

Fig. S4. The *F10* gene is expressed in Eastern brown snake (*Pseudonaja textilis*) venom gland tissue. One distinguishing feature between the *F10* and *vF10* paralogs is an 87-nucleotide deletion in exon 6 of the *vF10* gene. The corresponding nucleotide sequence in the *F10* gene (shown in orange in the sequence alignment) is absent in *vF10*. Taking advantage of this structural variant, we identified multiple short-read RNA-seq reads from *P. textilis* venom gland tissue that map to this *F10*-specific region (highlighted in orange), providing evidence that the hemostatic *F10* gene is also transcribed in the venom gland of *P. textilis*.

Pseudonaja textilis vF10a	MAPQLLLCLILTFLWSLPEAESNVFLKSKVANRFLQRTKRANSLVEEFKSGNIERECIEERCSKEEAREVFEDDEK
Pseudonaja textilis vF10b	MAPQLLLCLILTFLWSLPEAESNVFLKSKVANRFLQRTKRANSLVEEFKSGNIERECIEERCSKEEAREVFEDDEK
Pseudonaja textilis F10	MAPQLLLCLILTFLWSLPEAESNVFLKSKVANRFLQRTKRANSLFEEFKPGNIERECIEERCSKEEAREAFEDDEK
Pseudonaja textilis vF10a	TETFWNVYVDGDQCSSNPCHYRGICKDGIGSYTCTCLSGYEGKNCERVLYKSCR**VDNGNCWHFCK**SVQNDIQCSCA
Pseudonaja textilis vF10b	TETFWNVYVDGDQCSSNPCHYRGICKDGIGSYTCTCLSGYEGKNCERVLYKSCR**VDNGNCWHFCK**HVQNDIQCSCA
Pseudonaja textilis F10	TETFWNVYVDGDQCSSNPCHYGGTCKDGIGSYTCTCLSGYEGKNCEYVLYKSCR**VDNGDCWHFCK**PVQNGIQCSCA
Pseudonaja textilis vF10a	EGYLLGEDGHSCVAGGNFSCGRNIKTRNKREASLPDF-----------------------------VQSHNATLLK
Pseudonaja textilis vF10b	EGYLLGEDGHSCVAGGNFSCGRNIKTRNKREANLPDF-----------------------------VQSQNATLLK
Pseudonaja textilis F10	ESYLLGEDGHSCVAGGDFSCGRNIKTRNKREANLPDFQTDFSDDYDEIDENNFVETPTNFSGLVLTVQSQNATLLK
Pseudonaja textilis vF10a	**KSDNPSPDIRIVNGMDCK**LGECPWQAALVDDKKGVFCGGTILSPIYVLTAAHCINETETISVVVGEIDRSR**AETGP**
Pseudonaja textilis vF10b	**KSDNPSPDIRIVNGMDCK**LGECPWQAALVDEKEGVFCGGTILSPIYVLTAAHCINETETISVVVGEIDKSRIETGP
Pseudonaja textilis F10	**KSDNPSPDIR**VVNGTDCKLGECPWQALLLNDEGDGFCGGTILSPIYVLTAAHCINQTKYITVVVGEIDISSKKTGR
Pseudonaja textilis vF10a	**LLSVDKVYVHKKFVPPKKSQEFYEK**FDLVSYDYDIAIIQMKTPIQFSENVVPACLPTADFANQVLMKQDFGIVSGF
Pseudonaja textilis vF10b	LLSVDKIYVHK**KFVPPQK**AY----KFDLAAYDYDIAIIQMKTPIQFSENVVPACLPTADFANQVLMKQDFGIVSGF
Pseudonaja textilis F10	LHSVDKIYVHQKFVP-------------ATYDYDIAIIQLKTPIQFSENVVPACLPTADFANQVLMKQNFGIVSGF
Pseudonaja textilis vF10a	GGIFERGPNSKTLKVLKVPYVDRHTCMLSSNFPITPTMFCAGYDTLPQDACQGDSGGPHITAYRDTHFITGIVSWG
Pseudonaja textilis vF10b	GRIFEKGPKSKTLKVLKVPYVDRHTCMVSSETPITPNMFCAGYDTLPR**DACQGDSGGPHTTVYR**DTHFITGIVSSG
Pseudonaja textilis F10	GRTRERGKTSNTLKVVTLPYVDRHTCMLSSNFPITQNMFCAGYDTLPQDACQGDSGGPHITAYRDTHFITGIVSWG
Pseudonaja textilis vF10a	EGCARK**GRYGIYTK**LSKFIPWIKRIMR**QKLPSTESSTGRL**
Pseudonaja textilis vF10b	EGCARNGKYGIYTKLSKFIPWIKRIMR**QKLPSTESSTGRL**
Pseudonaja textilis F10	EGCAQTGKYGVYTKVSKFILWIKRIIRQKQPSTESSTGQL

Fig S5. Coagulation factor X protein is detected in Eastern brown snake (*Pseudonaja textilis*) venom. Amino acid sequence alignment of hypothetical *F10* gene translations with tryptic peptides shown in bold. Red residues indicate peptides unique to the venom paralogs (*vF10a* and/or *vF10b*), purple residues indicate peptides unique to the liver protein (*F10*), and black residues indicate peptides shared by all paralogs. Residues shown in grey correspond to the signal peptide and propeptide regions. Tryptic peptides corresponding to the hemostatic protein were detected in the venoms from three of seven individuals.

Oxyuranus microlepidotu F7b	MISCHFGVFLCLLLLASPIARTSVFLRHKEASNILQRAR**R**ANSFLEELKA**G**SIERECLE**E**KCS**W**EEAREIFQDDLR
transcript/829350	MISCHFGVFLCLLLLASPIARTSVFLRHKEASNILQRAR**R**ANSFLEELKA**G**SIERECLE**E**KCS**W**EEAREIFQDDLR
Oxyuranus microlepidotu F7a	MISCHFGVFLCLLLLASPIARTSVFLRHKEASNILQRAR**Q**ANSFLEELKA**E**SIERECLE**G**KCS**C**EEAREIFQDDLR
transcript/809936	MISCHFGVFLCLLLLASPIARTSVFLRHKEASNILQRAR**Q**ANSFLEELKA**E**SIERECLE**G**KCS**C**EEAREIFQDDLR
Oxyuranus microlepidotu F7b	TKEFW**A**IYIDPDQCDSNPCQNGGTCIDRPQDYFCICPPEHEGRNCEIGPEIKLKCF**FK**NGNCEQFCVDSPTTLRQC
transcript/829350	TKEFW**A**IYIDPDQCDSNPCQNGGTCIDRPQDYFCICPPEHEGRNCEIGPEIKLKCF**FK**NGNCEQFCVDSPTTLRQC
Oxyuranus microlepidotu F7a	TKEFW**E**IYIDPDQCDSNPCQNGGTCIDRPQDYFCICPPEHEGRNCEIGPEIKLKCF**LE**NGNCEQFCVDSPTTLRQC
transcript/809936	TKEFW**E**IYIDPDQCDSNPCQNGGTCIDRPQDYFCICPPEHEGRNCEIGPEIKLKCF**LE**NGNCEQFCVDSPTTLRQC
Oxyuranus microlepidotu F7b	SCAEGYRLASDQVSCIAEVDYPCGKIPVLAKRPNQQGRIVGGYDCPPGECPWQALITENGNEKCGGVLVAPSWIIS
transcript/829350	SCAEGYRLASDQVSCIAEVDYPCGKIPVLAKRPNQQGRIVGGYDCPPGECPWQALITENGNEKCGGVLVAPSWIIS
Oxyuranus microlepidotu F7a	SCAEGYRLASDQVSCIAEVDYPCGKIPVLAKRPNQQGRIVGGYDCPPGECPWQALITENGNEKCGGVLVAPSWIIS
transcript/809936	SCAEGYRLASDQVSCIAEVDYPCGKIPVLAKRPNQQGRIVGGYDCPPGECPWQALITENGNEKCGGVLVAPSWIIS
Oxyuranus microlepidotu F7b	AAHCFSRVRKENLRIRLGEYHLNHRDEGEQERRVDELIIHEKYSFKTVDNDIALLRLSAPVNFSEYVVPICLPPPR
transcript/829350	AAHCFSRVRKENLRIRLGEYHLNHRDEGEQERRVDELIIHEKYSFKTVDNDIALLRLSAPVNFSEYVVPICLPPPR
Oxyuranus microlepidotu F7a	AAHCFSRVRKENLRIRLGEYHLNHRDEGEQERRVDELIIHEKYSFKTVDNDIALLRLSAPVNFSEYVVPICLPPPR
transcript/809936	AAHCFSRVRKENLRIRLGEYHLNHRDEGEQERRVDELIIHEKYSFKTVDNDIALLRLSAPVNFSEYVVPICLPPPR
Oxyuranus microlepidotu F7b	FAADILNYVEFSTVSGWGRLLEGGSTSALLMRVEVPKIHKKECVRHTKFNITDNMFCAGYLDGSKDSCEGDSGGPH
transcript/829350	FAADILNYVEFSTVSGWGRLLEGGSTSALLMRVEVPKIHKKECVRHTKFNITDNMFCAGYLDGSKDSCEGDSGGPH
Oxyuranus microlepidotu F7a	FAADILNYVEFSTVSGWGRLLEGGSTSALLMRVEVPKIHKKECVRHTKFNITDNMFCAGYLDGSKDSCEGDSGGPH
transcript/809936	FAADILNYVEFSTVSGWGRLLEGGSTSALLMRVEVPKIHKKECVRHTKFNITDNMFCAGYLDGSKDSCEGDSGGPH
Oxyuranus microlepidotu F7b	VTEYKNTWYLTGLVSWGKGCAAIGTYGVYTKVIKYHQWLNNHMDS
transcript/829350	VTEYKNTWYLTGLVSWGKGCAAIGTYGVYTKVIKYHQWLNNHMDS
Oxyuranus microlepidotu F7a	VTEYKNTWYLTGLVSWGKGCAAIGTYGVYTKVIKYHQWLNNHMDS
transcript/809936	VTEYKNTWYLTGLVSWGKGCAAIGTYGVYTKVIKYHQWLNNHMDS

Fig S6. Full-length *F7* transcripts are present in inland taipan (*Oxyuranus microlepidotus*) venom gland tissue. Protein sequence alignment of the hypothetical protein translations encoded by the *F7a* and *F7b* paralogs of *O. microlepidotus*, together with representative full-length translated Iso-seq reads from venom gland tissue corresponding to each *F7* paralog. The alignment provides qualitative evidence that full-length *F7a and F7b* transcripts are expressed in venom gland tissue. Amino acid residues that differ between the *F7a* and *F7b* sequences are shown in bold.

Pseudonaja textilis F7a	MISCHFGVFLCLLLLASPIARTSVFLRHKEASNILQRARRANSFLEELKAESIEQECLEQKCSCEEAREIFQDDLR
Pseudonaja textilis F7b1	MVSCHFGVFLCLLLLASPIARTSVFLRHKEASNILQRARRANSFLEELKAGSIERECLEENCSWEEAREIFQDDMR
Pseudonaja textilis F7b2	MISWHFGVFLCLLLLASPIARTSVFLRHKEASNILQRARRANSFLEELKAGSIERECLEEKCSWEEAREIFQDDLR
Pseudonaja textilis F7a	TKEFCEIYIDPDQCDSNPCQNGGTCIDRPQDYFCICPPEHEGRNCEIGPEIKLKCFLENGNCEQFCVDSPTTLRQC
Pseudonaja textilis F7b1	TKEFWAIYIDPDQCDSNPCQNGGTCIDRPQDYFCICPPEHEGRNCEIGPETKLKCFFKNGNCEQFCVDSPTTLR**QC**
Pseudonaja textilis F7b2	TKEFWAIYIDPDQCDSNPCQNGGTCIDRPQDYFCICPPEHEGRNCEIGPETKLKCFFKNGNCEQFCVDSPTTLR**QC**
Pseudonaja textilis F7a	SCAEGYKLASDQVSCIAEVDYPCGKIPVLAKRPNQQGRIVGGYDCPPGECPWQALITENGNEKCGGVLVAPSWIIS
Pseudonaja textilis F7b1	**SCAEGYR**LASDQVSCIAEVDYPCGKIPVLAKRPNQQGRIVGGYDCPPGECPWQALITENGNEKCGGVLVAPSWVIS
Pseudonaja textilis F7b2	**SCAEGYR**LASDQVSCIAEVDYPCGKIPVLAKRPNQQGRIVGGYDCPPGECPWQALITENGNEKCGGVLVAPSWVIS
Pseudonaja textilis F7a	AAHCFSHLRKENLRIR**LGEYHLNHRDEGEQER**RVDELIIHENYSFKTVDNDIALLHLSAPVNFSEYVVPICLPPPR
Pseudonaja textilis F7b1	AAHCFSHLRKENLRIR**LGEYHLNHRDEGEQER**RVDELIIHEKYSFKTVDNDIALLRLSAPVNFSEYVVPICLPPPR
Pseudonaja textilis F7b2	AAHCFSRVHKENLRIR**LGEYHLNHRDEGEQER**RVDELIIHEKYSFKTVDNDIALLRLSAPVNFSEYVVPICLPPPR
Pseudonaja textilis F7a	FAADILNYVEFSTVSGWGRLLEGGSTSALLMRVEVPKIHKKECVRHTKFNITDNMFCAGYLDGSK**DSCEGDSGGPH**
Pseudonaja textilis F7b1	FAADVLNYVEFSTVSGWGRLLEGGSTSALLMRVQVPKIHKKECVQHTKFNITDNMFCAGYLDGSK**DSCEGDSGGPH**
Pseudonaja textilis F7b2	FAADVLNYVEFSTVSGWGRLLEGGSTSALLMRVQVPKIHKKECVRHTKFNITDNMFCAGYLDGSK**DSCEGDSGGPH**
Pseudonaja textilis F7a	**VTEYK**NTWYLTGLVSWGKGCAAIGTYGVYTKVIKYHQWLNNHMDS
Pseudonaja textilis F7b1	**VTEYK**NTWYLTGLVSWGKGCAAIGTYGVYTKVIKYHQWLNNHMDS
Pseudonaja textilis F7b2	**VTEYK**NTWYLTGLVSWGKGCAAIGTYGVYTKVIKYHQWLNNHMDS

Fig S7. Coagulation factor VII proteins are detected in Eastern brown snake (*Pseudonaja textilis*) venom. Amino acid sequence alignment of hypothetical *F7* gene translations with tryptic peptides shown in bold. Residues shown in grey correspond to the signal peptide and propeptide regions. Tryptic peptides were detected in venom from three of seven individuals.

	1 … 85
P. textilis vF5 (short)	MGRYSVSPVPKCLLLMFLGWSGLKYYQVNAAQLREYHIAAQLEDWDYNPQPEELSRLSESDLTFKKIVYREYELDFKQEKPRDAL
O. microlepidotus vF5 (short)	MGRYSVSPVPKCLLLMFLGWSGLKYYQVNAAQLREYRIAAQLEDWDYNPQPEELSRLSESELTFKKIVYREYELDFKQEKPRDEL
P. textilis F5 (short)	MGRYSVSPVPKCLLLMFLGWSGLKYYQVNAAQLREYRIAAQLEDWDYNPQPEELSRLSESDLTFKKIVYREYELDFKQEKPRDAL
O. microlepidotus F5 (short)	MGRYSVSPVPKCLLLMFLGWSGLKYYQVNAAQLREYRIAAQLEDWDYSPQPEELSRLSESELTFKKIVYREYELDFKQEKPRDEL
P. textilis F5	MGRYSVSPVPKCLLLMFLGWSGLKYYQVNAAQLREYRIAAQLEDWDYNPQPEELSRLSESDLTFKKIVYREYELDFKQEKPRDAL
O. microlepidotus F5	MGRYSVSPVPKCLLLMFLGWSGLKYYQVNAAQLREYRIAAQLEDWDYSPQPEELSRLSESELTFKKIVYREYELDFKQEKPRDEL
N. scutatus F5	MGRYSVSPVPKCLLLMFLGWSGLKYYQVNAAQLREYRIAAQLEDWDYNLQPEELSRLSESDRTFQKIVYREYELDFKQEKPRDEL
B. candidus F5	MGRYSVSPVSKCLLLMFLGWSGLKNYQVNAAQLREYRIAAQLEDWDYNLQPEGLSRLSESDRTFRKIVYREYELDFKQEKPRDKL
N. helvetica F5	MGRYSVSPIPKCLLLVFLGWSGLKYYQVNAAQLREYHIAAQVEDWDYNLQPGGLSRLSESDLAFKKIVYREYEVDFKQEKTRDEL

	86 … 170
P. textilis vF5 (short)	SGLLGPTLRGEVGDSLIIYFKNFATQPVSIHPQSAVYNKWSEGSSYSDGTSDVERLDDAVPPGQSFKYVWNITAEIGPKKADPPC
O. microlepidotus vF5 (short)	SGLLGPTLRGEVGDILIIYFKNFATQPVSIHPQSAVYNKWSEGSSYSDGTSDVERLDDAVPPGQSFKYVWNITAEIGPKKADPPC
P. textilis F5 (short)	SGLLGPTLRGEVGDILIIYFKNFATQPVSIHPQSAVYNKWSEGSSYSDGTSDVERLDDAVPPGQSFKYVWNITAEIGPKKADPPC
O. microlepidotus F5 (short)	SGLLGPTLRGEVGDILIIYFKNFATQPVSIHPQSAVYNKWSEGSSYSDGTSDVERLDDAVPPGQSFKYVWNITAEIGPKKADPPC
P. textilis F5	SGLLGPTLRGEVGDILIIYFKNFATQPVSIHPQSAVYNKWSEGSSYSDGTSDVERLDDAVPPGQSFKYVWNITAEIGPKKADPPC
O. microlepidotus F5	SGLLGPTLRGEVGDILIIYFKNFATQPVSIHPQSAVYNKWSEGSSYSDGTSDVERLDDAVPPGQSFKYVWNITAEIGPKKADPPC
N. scutatus F5	SGLLGPTLRGEVGDILIIYFKNFATKPVSIHPQSAVYNKWSEGSSYSDGTSDVERLDDAVPPGQSFKYVWNITAEIGPKKADPPC
B. candidus F5	SGLLGPTLRGEVGDILIIYFKNFATQPVSIHPQSAVYNKWSEGSSYSDGTSDMERLDNAVPPGQAFKYVWNITAEIGPKKADPPC
N. helvetica F5	SGLLGPTLRGEVGDVLVIYFKNFATQPVSIHPQSAVYNKWSEGASYSDGTSDVEKLDDAVPPDHSFKYVWNITAEIGPKETDPPC

	171 … 255
P. textilis vF5 (short)	LTYAYYSHVNMVRDFNSGLIGALLICKEGSLNANGSQKFFNREYVLMFSVFDESKNWYRKPSLQYTINGFANGTLPDVQACAYDH
O. microlepidotus vF5 (short)	LTYAYYSHVNMVRDFNSGLIGALLICKEGSLNANGAQKFFNREYVLMFSVFDESKNWYRKPSLQYTINGFANGTLPDVQACAYDH
P. textilis F5 (short)	LTYAYYSHVNMVRDFNSGLIGALLICKEGSLNANGAQKFFNREYVLMFSVFDESKNWYRKPSLQYTINGFANGTLPDVQACAYDH
O. microlepidotus F5 (short)	LTYSYYSHVNMVRDFNSGLIGALLICKEGSLNANGAQKFFNREYVLMFSVFDESKNWYRKPSLQYTINGFANGTLPDVQACAYDH
P. textilis F5	LTYAYYSHVNMVRDFNSGLIGALLICKEGSLNANGAQKFFNREYVLMFSVFDESKNWYRKPSLQYTINGFANGTLPDVQACAYDH
O. microlepidotus F5	LTYSYYSHVNMVRDFNSGLIGALLICKEGSLNANGAQKFFNREYVLMFSVFDESKNWYRKPSLQYTINGFANGTLPDVQACAYDH
N. scutatus F5	LTYAYYSHVNMVRDFNSGLIGALLICKEGSLNANGTQKLFNREYVLMFSVFDESKNWYRKPSLQYTINGFANGTLPDVQACAYDH
B. candidus F5	LTYAYYSHVNMVQDFNSGLIGALLICKKGSLTANGTQKLFNREYVLMFSVFDESKNWYKKPSLQYTINGFANGTLPDIQACAYDR
N. helvetica F5	LTYAYYSHVNMVRDFNSGLIGALLICKEGSLNADGTQKLFNREYVLMFAVFDESKNWYKKPSLQYTINGFANGTLPDIQACAYDR

	256 … 340
P. textilis vF5 (short)	ISWHLIGMSSSPEIFSVHFNGQTLEQNHYKVSTINLVGGASVTADMSVSRTGKWLISSLVAKHLQAGMYGYLNIKDCGNPDTLTR
O. microlepidotus vF5 (short)	ISWHLIGMSSSPEIFSVHFNGQTLEQNHYKVSTINLVGGASVTANMSVSRTGKWLISSLVAKHLQAGMYGYLNIKDCGHPNTLTR
P. textilis F5 (short)	ISWHLIGMSSSPEIFSVHFNGQTLEQNHYKVSTINLVGGASVTANMSVSRTGKWLISSLVAKHLQAGMYGYLNIKDCGNPDTLTR
O. microlepidotus F5 (short)	ISWHLIGMSSSPEIFSVHFNGQTLEQNHYKVSTINLVGGASVTANMSVSRTGKWLISSLVAKHLQAGMYGYLNIKDCGNPDTLTR
P. textilis F5	ISWHLIGMSSSPEIFSVHFNGQTLEQNHYKVSTINLVGGASVTANMSVSRTGKWLISSLVAKHLQAGMYGYLNIKDCGNPDTLTR
O. microlepidotus F5	ISWHLIGMSSSPEIFSVHFNGQTLEQNHYKVSTINLVGGASVTANMSVSRTGKWLISSLVAKHLQAGMYGYLNIKDCGNPDTLTR
N. scutatus F5	ISWHLIGMSSSPEIFSVHFNGQTLEQNHYKVSTINLIGGASVTAKMSVSRTGKWLISSLVAKHLQAGMYGYLNIKDCGNPDTLTR
B. candidus F5	ISWHLIGMSSSPEIFSVHFNGQTLEQNNYKVSTINLVGGASVTANMSVSRTGKWLISSLVAKHLQAGMYGYLNIKDCGKSDTLTR
N. helvetica F5	ISWHLIGMSSSPEIFSVHFNGQTLEENHYKVSTINLVGGASVTANMSVSMTGKWLISSLVAKHLQAGMYGYLNIKDCGKPDTLTK

	341 … 425
P. textilis vF5 (short)	KLSFRELMKIKNWEYFIAAEEITWDYAPEIPSSVDRRYKAQYLDNFSNFIGKKYKKAVFRQYEDGNFTKPTYAIWPKERGILGPV
O. microlepidotus vF5 (short)	KLSFRELRRIMNWEYFIAAEEITWDYAPEIPSSVDRRYKAQYLDNFSNFIGKKYKKAVFRQYEDGNFTKPTYAIWPKERGILGPV
P. textilis F5 (short)	KLSFRELRRIMNWEYFIAAEEITWDYAPEIPSSVDRRYKAQYLDNFSNFIGKKYKKAVFRQYKDSNFTKPTYAIWPKERGILGPV
O. microlepidotus F5 (short)	KLSFRELRRIMNWEYFIAAEEITWDYAPEIPSSVDRRYKAQYLDNFSNFIGKKYKKAVFRQYKDGNFTKPTYAIWPKERGILGPV
P. textilis F5	KLSFRELRRIMNWEYFIAAEEITWDYAPEIPSSVDRRYKAQYLDNFSNFIGKKYKKAVFRQYKDSNFTKPTYAIWPKERGILGPV
O. microlepidotus F5	KLSFRELRRIMNWEYFIAAEEITWDYAPEIPSSVDRRYKAQYLDNFSNFIGKKYKKAVFRQYKDGNFTKPTYAIWPKERGILGPV
N. scutatus F5	KLSFRELKMIKNWEYFIAAEEITWDYAPEIPRSVDRRYKAQYLDNFSNFIGKKYKKAVFRQYKDSSFSKPTYAIWPKERGILGPV
B. candidus F5	KLSFKELRMIKNWEYFIAAEEITWDYAPEIPSTVDRRYKAQYLDNFSNFIGKKYKKAVFRQYKDSSFTKPTYAIWPKERGILGPV
N. helvetica F5	RLTFKELRMIKNWDYFIAAEEITWDYAPEIPSSVDRRYKAQYLDNFSNFIGKKYKKAVFRQYKDSSFSKPTYATWPKERGILGPV

	426 … 510
P. textilis vF5 (short)	IKAKVRDTVTIVFKNLASRPYSIYVHGVSVSKDAEGAIYPSDPKENITHGKAVEPGQVYTYKWTVLDTDEPTVKDSECITKLYHS
O. microlepidotus vF5 (short)	IKAKVRDTVTIVFKNLASRPYSIYVHGVSVSKDAEGAIYPSDPKENITHGKAVEPGQVYTYKWTVLDTDEPTVKDSECITKLYHS
P. textilis F5 (short)	IRAKVRDTISIVFKNLASRPYSIYVHGVSVSKDAEGAIYPSDPKENITHGKAVEPGQVYTYKWTVLDTDEPTVKDSECITKLYHS
O. microlepidotus F5 (short)	IRAKVRDTISIVFKNLASRPYSIYVHGVSVSKDAEGAIYPSDPKENITHGKAVEPGQVYTYKWTVLDTDEPTVKDSECITKLYHS
P. textilis F5	IRAKVRDTISIVFKNLASRPYSIYVHGVSVSKDAEGAIYPSDPKENITHGKAVEPGQVYTYKWTVLDTDEPTVKDSECITKLYHS
O. microlepidotus F5	IRAKVRDTISIVFKNLASRPYSIYVHGVSVSKDAEGAIYPSDPKENITHGKAVEPGQVYTYKWTVLDTDEPTVKDSECITKLYHS
N. scutatus F5	IRAEVRDTISIVFKNLASRPYSIYVHGVSVSKDAEGAIYPSDPKENITHGKAVEPGQVYTYKWTVLDTDEPTVKDSQCITKLYHS
B. candidus F5	IRAEVRDTISIVFKNLASRPYSIYVHGVSVSKDAEGAIYPSDPKENITHGKAVEPGHIYTYKWTVLDTDEPTVKDSQCITKLYHS
N. helvetica F5	IRAGVRDTISIVFKNLASRPYSIYVHGVSVSKDAEGAVYPSDSKENITQGKAVEPGDVYTYKWTVLDTDEPTAKDSQCITKLYHS

	511 … 595
P. textilis vF5 (short)	AVDMTRDIASGLIGPLLVCKHKALSVKGVQNKADVEQHAVFAVFDENKSWYLEDNIKKYCSNPSAVKKDDPKFYKSNVMYTLNGY
O. microlepidotus vF5 (short)	AVDMTRDIASGLIGPLLVCKLKALSVKGVQNKADVEQHAVFAVFDENKSWYLEDNIKKYCSNPSSVKKDDPKFYKSNVMYTLNGY
P. textilis F5 (short)	AVDMTRDIASGLIGPLLVCKHKALSVKGVQNKADVEQHAVFAVFDENKSWYLEDNIKKYCSNPSTVKKDDPKFYKSNVMYTLNGY
O. microlepidotus F5 (short)	AVDMTRDIASGLIGPLLVCKRKALSVKGVQNKADVEQHAVFAVFDENKSWYLEDNIKKYCSNPSSVKKDDPKFYKSNVMYTLNGY
P. textilis F5	AVDMTRDIASGLIGPLLVCKHKALSVKGVQNKADVEQHAVFAVFDENKSWYLEDNIKKYCSNPSTVKKDDPKFYKSNVMYTLNGY
O. microlepidotus F5	AVDMTRDIASGLIGPLLVCKRKALSVKGVQNKADVEQHAVFAVFDENKSWYLEDNIKKYCSNPSSVKKDDPKFYKSNVMYTLNGY
N. scutatus F5	AVDMTRDIASGLIGPLLVCKRKALNSKGVQDKADVEQHAVFAVFDENKSWYLEDNIKKYCSNSSTVKKDDPKFYKSNVMYTLNGY
B. candidus F5	AVDMTRDIASGLIGPLLVCKRKALDSKGVQKKADVEQHAVFAVFDENKSWYLEDNIKKYCSNSSTVKKDDPKFYKSNVMYTLNGY
N. helvetica F5	AVDMTRDIASGLIGPLLVCKRKALDNRGVQKKADVEQHAIFAVFDENKSWYLDDNIKKYSSNFSTVKKDDPKFYKSNVMYTLNGY

```
                                 596                                                                             680
P. textilis vF5 (short)          ASDRTEVLRFHQSEVVQWHLTSVGTVDEIVPVHLSGHTFLSKGKHQDILNLFPMSGESATVTMDNLGTWLLSSWGSCEMSNGMRL
O. microlepidotus vF5 (short)    ASDRTEVLGFHQSEVVQWHLTSVGTVDEIVPVHLSGHTFLSKGKHQDILNLFPMSGESATVTMDNLGTWLLSSWGSCEMSNGMRL
P. textilis F5 (short)           ASDRTEVLGFHQSEVVEWHLTSVGTVDEIVPVHLSGHTFLSKGKHQDILNLFPMSGESATVTMDNLGTWLLSSWGSCEMSNGMRL
O. microlepidotus F5 (short)     ASDRTEVLGFHQSEVVEWHLTSVGTVDEIVPVHLSGHTFLSKGKHQDILNLFPMSGESATVTMDNLGTWLLSSWGSCEMSNGMRL
P. textilis F5                   ASDRTEVLGFHQSEVVEWHLTSVGTVDEIVPVHLSGHTFLSKGKHQDILNLFPMSGESATVTMDNLGTWLLSSWGSCEMSNGMRL
O. microlepidotus F5             ASDRTEVLGFHQSEVVEWHLTSVGTVDEIVPVHLSGHTFLSKGKHQDILNLFPMSGESATVTMDNLGTWLLSSWGSCEMSNGMRL
N. scutatus F5                   ASDRTDVLGFRQSEIVEWHLTSVGTVDEIVPVHLSGHTFLSKGKHQDILNLFPMSGESATVTMDNLGTWLLSSWGSREMSNGMRL
B. candidus F5                   ASDRTDVLGFRQSEIVEWHLTSVGTVDEIVPVHLSGHTFLSKGKHQDILNLFPMRGESATVTMDNLGTWLLSSWGSREMSNGMRL
N. helvetica F5                  ASDRTEVLGFHQSEIVEWHLTSVGTMDEIIPVHLSGHTFLSKGKHQDILNLFPMSGESATVTMDNLGTWLLSSWGSREMSNGMRL
```

```
                                 681                                                                             765
P. textilis vF5 (short)          RFLDANYDDEDEGNEEEEEDDGDIFADI--FIPSEVVKK---KEEVPVNFVPDPESDALAKELGLIDDEGNPIIQPRREQTEDDE
O. microlepidotus vF5 (short)    RFLDANYDDEDEGNEEEEEDDGDIFADI--FSPPEVVKK---KEEVPVNFVPDPESDALAKELGLLDDEDNP-EQSRSEQTEDDE
P. textilis F5 (short)           RFLDANYDDEDEGNEEEEEDDGDIFADI--FIPPEVVKK---KEKDPVNFVSDPESDKIAKELGLLDDEDNQ-EESHNVQTEDDE
O. microlepidotus F5 (short)     RFLDANYDDEDEGNEEEEEDDGDIFADI--FSPPEVVKK---KEKDPVNFVSDPESDKIAKELGLLDDEDNQ-EESHNEQTEDDE
P. textilis F5                   RFLDANYDDEDEGNEEEEEDDGDIFADI--FIPPEVVKK---KEKDPVNFVSDPESDKIAKELGLLDDEDNQ-EESHNVQTEDDE
O. microlepidotus F5             RFLDANYDDEDEGNEEEEEDDGDIFADI--FSPPEVVKK---KEKDPVNFVSDPESDKIAKELGLLDDEDNQ-EESHNEQTEDDE
N. scutatus F5                   RFLDAKHDDEDEGNEKEEEHYGGIVANIISGGPSEVVKK---KEKDPRNVVSDSEMDKIAKELGLLDDEDNQ-KESSNEQTEDDE
B. candidus F5                   RFLDANYDDEDEGNEKEEEEDGDIVANIVSFSSPDVIKE---KRKDSKSVVSDPETDKIAKELGLLDDEDNQ-KESSNEQIEDDE
N. helvetica F5                  RFLDANYDDEDDRDEK-EEDYDDIVANIVSFDPSEVTEKKEEKEKDLRKVVSDPESDEIAKQLGLLDDEDST-YKSSNEQAEDDE
```

```
                                 766                                                                             850
P. textilis vF5 (short)          EQLMKASMLGLRSFKGSVA--EEELKHTALALEEDAHAS----------------------------------------------
O. microlepidotus vF5 (short)    EQLMIASMLGLRSFKGSVA--EEELKHTALALEEDAHAS----------------------------------------------
P. textilis F5 (short)           EQLMIATMLGFRSFKGSVA--EEELNLTALALEEDAHAS----------------------------------------------
O. microlepidotus F5 (short)     EQLMIATMLGLRSFKGSVA--EEELNLTALALEEDAHAS----------------------------------------------
P. textilis F5                   EQLMIATMLGFRSFKGSVA--EEELNLTALALEEDAHASGELSLNTHLVLINSTDAMRGNEELGTQNNSYVNASETIYANYTSST
O. microlepidotus F5             EQLMIATMLGLRSFKGSVA--EEELNLTALALEEDAHASGELSLNTHLVLINSTDAMRGNEELGTRNNSYVNASETIYANYTSST
N. scutatus F5                   EQLMIASMLGLRSFNGSVA--EEELNLTALALEEDAHTSGELSLNTHLVLINSTDAMRGNEELGTQNNSYVNASETIYANYTSST
B. candidus F5                   EQLMIASMLGLRTFNGSVA-EEEELNLTALALQEDAHTSGDVSLNTHLVLINSTDAMHGNEELGTQNNSYVTASETIYANYTSST
N. helvetica F5                  EQLMIASMLGLRSFNGSVAEEEEEELNLTALAIQEDADASGDVSLNDYIALNNSSDAMLGNGELGTQNNSYVTASETIYANCTSLA
```

```
                                 851                                                                             935
P. textilis vF5 (short)          -------------------------------------------------------------------------------------
O. microlepidotus vF5 (short)    -------------------------------------------------------------------------------------
P. textilis F5 (short)           -------------------------------------------------------------------------------------
O. microlepidotus F5 (short)     -------------------------------------------------------------------------------------
P. textilis F5                   GEILISNITAMIGILLSKRSQNEENITQISDSYKEMLAAEYLKAGRGLQSKDIEDLMSPEIIPSLKEQEENTFEKDIWDKKGERQ
O. microlepidotus F5             GEILISNITAMIGILLSKKSQNEENITQISDSYKQMLAAEYLKAGRGLQSQDIEDLMSPEIIPSLKEQEENTFEKDIWDKKGERQ
N. scutatus F5                   GEILISNITAMIGILLSKKSQNEENITQISDSYKQMLAAEYLKAGRGLQSKDIEDLMSPEIIPSLKEQEENTFEKDIWDKQGERQ
B. candidus F5                   GEILISNITEMIGILLSKKSQNEENITQILDSYKQMLAAEYLKAGRGLQSKDIEDLISLEIIPSLKKQEENTFEKDIWDKKGERQ
N. helvetica F5                  GEILTSNITAVIDILLSKISQNEENITQISDSCKQFLAAEYLKTDRGPQSKDIEDLMSLEIISSMKEQEENTFEKYIWDKKRETQ
```

```
                                 936                                                                            1020
P. textilis vF5 (short)          -------------------------------------------------------------------------------------
O. microlepidotus vF5 (short)    -------------------------------------------------------------------------------------
P. textilis F5 (short)           -------------------------------------------------------------------------------------
O. microlepidotus F5 (short)     -------------------------------------------------------------------------------------
P. textilis F5                   ILTEATLHALKEMHALFFYVQQKRNLSAFNITSEGMKFLPSTENDSTIDNSKANYFSYDYDYDYKEEEATAAANVEFSKVIINTI
O. microlepidotus F5             ILTEATLHALKEMHALFFYVQQKRNLSAFNITSEDMKFLPSTENDSTIDNSKANYFSYDYDYDYKEEEATAAANVEFSKVIINTI
N. scutatus F5                   ILTEATLHALKEMHALFFYVQQKRNLSAFNITSEDMKFLSSTENDSTTDNSKANYFSYDYDYDYKEEEATAAANVEFSKVITNTI
B. candidus F5                   VLSEATLHALKEMYALFFYVQQKRNISAFNITNEDMKFLSSTENDSTLDNSKANYFSYDYDYDYKGGEATAAANVEFPKVITNAT
N. helvetica F5                  VLTETTLYALKEMYALFFHVQQKRNLSAFNITSEDMKFLPSTENDSTLDDSKANYFPYEYDYDYK-EEATAAANVEFSKVIMNIT
```

```
                                 1021                                                                           1105
P. textilis vF5 (short)          -------------------------------------------------------------------------------------
O. microlepidotus vF5 (short)    -------------------------------------------------------------------------------------
P. textilis F5 (short)           -------------------------------------------------------------------------------------
O. microlepidotus F5 (short)     -------------------------------------------------------------------------------------
P. textilis F5                   SDDKKTVENNRSEVNLVSVPSIMPQPENLTSDATLDNFVSTTISKTTKTWNSSSHQRQKCLPKNTDMEW----KKENYDTLADIP
O. microlepidotus F5             SDDKKTVENNRSEVNLVSVPSIMPQPENLTSDATLDNFVSTTISKTTKAWNSSSHQRQKCLPKNTDMEW----KKENYDTLADIP
N. scutatus F5                   SDDKKTVENNGSEVNLASVPSIMPQPENLTSDATLDNFVSTTILKTTKTWNSSSHQRQKCLPKNTDMEW----KKENYDTLADIT
B. candidus F5                   SADKKTVENNGSELNLVSVPSIMPQPENLTSDATLDNFVSTTISKTTKTWNSSSHQKQKCLPKNTGMGW----KKENYDTLADIT
N. helvetica F5                  SADKETVENNGSEPNLVSVPSIMPQSENLTSNATLDNFVSTTISNSTKTWKRSSPQRQKCFPKNTDKEWNVVDKKENYDILADIT
```

```
                                 1106                                                                           1190
P. textilis vF5 (short)          -------------------------------------------------------------------------------------
O. microlepidotus vF5 (short)    -------------------------------------------------------------------------------------
P. textilis F5 (short)           -------------------------------------------------------------------------------------
O. microlepidotus F5 (short)     -------------------------------------------------------------------------------------
P. textilis F5                   NDVDNKFQNSSRNASFYQENAMFPGKRKEKTDNSNGQLELGQPGITNKHDSQKDENCTINISQSFIKIQRKKKKPKVLTPRTSKH
O. microlepidotus F5             NDVDNKFQNSSRNASFYQENAMFPGKRKEKTDNSNGQLELGQPGITNKHDSQKDENCTINISQSFIKIQRKKKKPKVLTPRTSKH
N. scutatus F5                   NDVDNKFQNSSRNASFYQENAMFPGKRKEKTDNSNGQLELGQPGITNKHDSQKDENCTINISQSFIKIQRKKKKPKVLTPRASKH
B. candidus F5                   NDGDNKFQNSSRNASFYQENAMFPDKRKEKTDNSNGQLELGQPGITNKHDAQKDENCTINISQSFIKIQRKKKKPKVLTPRTSKP
N. helvetica F5                  NDVDNKFQNSSRNASFYQENALFPGKRKEKTDNSNGQLELGQPGINNKNDAQKDENCTINISQPFVKIQRKKKKPNVLAPRTFKP
```

	1191 … 1275
P. textilis vF5 (short)	**--DPRIDSN**
O. microlepidotus vF5 (short)	**--DPRIDSN**
P. textilis F5 (short)	**--DPRIDSN**
O. microlepidotus F5 (short)	**--DPRIDSN**
P. textilis F5	**LELPRNLNNSQLSNKPNHTKLLNEAKLTRKQDKLIVTIGLPVEDGDYQEYDIDNTDNDRSSSGSFEYELMYYNNPYTTDPRIDSN**
O. microlepidotus F5	**LELPRNLNNSQLSNKPNHTKLLNEAKLTRKQDKFIVTIGLPVEDGDYQEYEIDNTDNDKSSSGSFEYELMYYNNPYTTDPRIDSN**
N. scutatus F5	**LELPRNLNHSQLSNKPNHTKLLNEAKLTRKQDK-LVTIGLPVEDGDYQEYEIDNTDNDKSSSGSFEYELMYYDNPYTTDPRIDSN**
B. candidus F5	**LDLPRNLNNSQFSNKPNHTKLLNEAKLTRKQDKPIVTIGLPVEDGDYQEYEIDNIDNDQSSSGSFEYELMFYDNPYTTDSRLDST**
N. helvetica F5	**LELPANLNNSQLSNKLNHTKLLNEAKLTQKQDKPIITIGLPVEDGGYQEYEIDNTDNDESSSGSFEYELMYYDNPYTTDSRLDSN**

	1276 … 1360
P. textilis vF5 (short)	**SARNPDDIAGRYLR**TINRGNKRRYYIAAEEVLWDYSPIGKSQVRSRAAKTTFKKAIFRSYLDDTFQTPSTGGEYEKHLGILGPII
O. microlepidotus vF5 (short)	**SARNSDDIAGRYLR**TINRRNKRRYYIAAEEVLWDYSPIGKSQVRSLPAKTTFKKAIFRSYLDDTFQTPSTGGEYEKHLGILGPII
P. textilis F5 (short)	**SARNPDDIAGRYLR**TINRGNKRRYYIAAEEVLWDYSPIGKSQVRSRAAKTTFKKAIFRSYLDDTFQTPSTGGEYEKHLGILGPII
O. microlepidotus F5 (short)	**SARNPDDIAGRYLR**TINRGNKRRYYIAAEEVLWDYSPIGKSQVRSRAAKTTFKKAIFRSYLDDTFQTPSTGGEYEKHLGILGPII
P. textilis F5	**SARNPDDIAGRYLR**TINRGNKRRYYIAAEEVLWDYSPIGKSQVRSRAAKTTFKKAIFRSYLDDTFQTPSTGGEYEKHLGILGPII
O. microlepidotus F5	**SARNPDDIAGRYLR**TINRGNKRRYYIAAEEVLWDYSPIGKSQVRSRAAKTTFKKAIFRSYLDDTFQTPSTGGEYEKHLGILGPII
N. scutatus F5	**SARNPDDIARHYLR**TTNSGNKRMYYIAAEEVLWDYSPIRKSQVRSHPAKTTFKKAIFRSYLDDTFQTPSTGGEYEKHLGILGPII
B. candidus F5	**SARNPDDIAGRYMR**TINRGNIRKYYIAAEEVLWDYSPIRKSQVRSRPVKTTFKKAIFRSYLDDTFQTPSTGGEYEKHLGILGPII
N. helvetica F5	**IARNPDDIAGRYLR**TINRGNIRRYYIAAEEIVWDYSPFRKSTVRSRPLRTTFKKAIFRSYLDDTFQTPSPVGEYEKHLGILGPII

	1361 … 1445
P. textilis vF5 (short)	RAEVDDVIEIQFRNLASRPYSLHAHGLLYEKSSEGRSYDDKSPELFKKDDAIMPNGTYTYVWQVPPRSGPTDNTEKCKSWAYYSG
O. microlepidotus vF5 (short)	RAEVDDVIEVQFRNLASRPYSLHAHGLLYEKSSEGRSYDDNSPELFKKDDAIMPNGTYTYVWQVPPRSGPTDNTEKCKSWAYYSG
P. textilis F5 (short)	RAEVDDVIEVQFRNLASRPYSLHAHGLLYEKSSEGRSYDDKSPELFKKDDAIMPNGTYTYVWQVPPRSGPTDNTEKCKSWAYYSG
O. microlepidotus F5 (short)	RAEVDDVIEVQFRNLASRPYSLHAHGLLYEKSSEGRSYDDKSPELFKKDDAIMPNGTYTYVWQVPPRSGPTDNTEKCKSWAYYSG
P. textilis F5	RAEVDDVIEVQFRNLASRPYSLHAHGLLYEKSSEGRSYDDKSPELFKKDDAIMPNGTYTYVWQVPPRSGPTDNTEKCKSWAYYSG
O. microlepidotus F5	RAEVDDVIEVQFRNLASRPYSLHAHGLLYEKSSEGRSYDDKSPELFKKDDAIMPNGTYTYVWQVPPRSGPTDNTEKCKSWAYYSG
N. scutatus F5	RAEVDDVIEVQFKNLASRPYSLHAHGLLYDKSSEGRSYDDQSPELFKKDEAIMPNGTYTYVWHVPQRSGPTDNTEKCKSWAYYSG
B. candidus F5	RAEVDDVIQVQFRNLASRPYSLHAHGLLYEKSSEGRSYDDQSPEFFKMDDAIMPNGTYTYVWQVTKRSGPTDNTEKCKSWAYYSG
N. helvetica F5	RAEVDDVIQVQFRNLASRPYSLHAHGLLYEKSSEGRSYDDHSPEFFKKDDAIMPNSTYTYVWQVTKRAGPTDDTEKCKTWAYYSG

	1446 … 1530
P. textilis vF5 (short)	VNPEKDIHSGLIGPILICQKGMIDKYNRTIDIREFVLFFMVFDEEKSWYFPKSDKSTCEEKLIGVQSLHTFPAINGIPYQLQGLT
O. microlepidotus vF5 (short)	VNPEKDIHSGLIGPILICQKGMIDKYNRTIDIREFVLFFMVFDEEKSWYFPKSDKSTCEEKLIGVQSHHTFPAINGIPYQLQGLM
P. textilis F5 (short)	VNPEKDIHSGLIGPILICQKGMIDKYNRTIDIREFVLFFMVFDEEKSWYFPKSDKSTRAEKLIGVQSRHTFPAINGIPYQLQGLT
O. microlepidotus F5 (short)	VNPEKDIHSGLIGPILICQKGMIDKYNRTIDIREFVLFFMVFDEEKSWYFPKSDKSTRAEKLIGVQSRHTFPAINGIPYQLQGLM
P. textilis F5	VNPEKDIHSGLIGPILICQKGMIDKYNRTIDIREFVLFFMVFDEEKSWYFPKSDKSTRAEKLIGVQSRHTFPAINGIPYQLQGLT
O. microlepidotus F5	VNPEKDIHSGLIGPILICQKGMIDKYNRTIDIREFVLFFMVFDEEKSWYFPKSDKSTRAEKLIGVQSRHTFPAINGIPYQLQGLM
N. scutatus F5	VNPEKDIHSGLIGPILICQKGMIDKYNKTIDIREFVLFFMVFDEERSWYFAKSDKRTRAEKLIGVQSRHTFPAINGIPYQLQGLK
B. candidus F5	VNPEKDIHSGLIGPILICQKGMIDEYNRPIDIREFVLFFMVFDEERSWYFAKSDKRTRAEKLIGVQSRHTFPAINGIPYQLQGLK
N. helvetica F5	VNPEKDIHSGLIGPILICHKHMIDEYNRPIDIREFVLLFMVFDEERSWYFEKSDKRTRTERLLGVQSRHTFPAINGIPYQLQGLK

	1531 … 1615
P. textilis vF5 (short)	MYKDENVHWHLLNMGGPKDIHVVNFHGQTFTEEGREDNQLGVLPLLPGTFASIKMKPSKIGTWLLETEVGENQERGMQALFTVID
O. microlepidotus vF5 (short)	MYKDENVHWHLLNMGGPKDIHVVNFHGQTFTEEGREDNQLGVLPLLPGTFASIKMKPSKIGTWLLETEVGENQERGMQALFTVID
P. textilis F5 (short)	MYKDENVHWHLLNMGGPKDIHVVNFHGQTFTEEGREDNQLGVLPLLPGTFASIKMKPSKIGTWLLETEVGENQERGMQALFTVID
O. microlepidotus F5 (short)	MYKDENVHWHLLNMGGPKDIHVVNFHGQTFTEEGREDNQLGVLPLLPGTFTSIKMKPSKIGTWLLETEVGENQERGMQALFTVID
P. textilis F5	MYKDENVHWHLLNMGGPKDIHVVNFHGQTFTEEGREDNQLGVLPLLPGTFASIKMKPSKIGTWLLETEVGENQERGMQALFTVID
O. microlepidotus F5	MYKDENVHWHLLNMGGPKDIHVVNFHGQTFTEEGREDNQLGVLPLLPGTFTSIKMKPSKIGTWLLETEVGENQERGMQALFTVID
N. scutatus F5	MYKDENVHWHLLNMGGPKDIHVVKFHGQTFTEEGMEDNQLGVLPLLPGTFTSIKMKPSKIGTWLLETEVGENRERGMQALFTVID
B. candidus F5	MYKDENVHWHLLNMGGPKDIHVVNFHGQTFTEEGREDNQLGVLPLLPGTFTSIKMKPSKIGTWLLETEVGEYQERGMQALFTVID
N. helvetica F5	MYKEENVHWHLLNMGGPKDIHVVHFHGQTFTEEGREDNQLGVLPLLPGTFTTIKMKPSKIGTWLLKTEVGEYQERGMKALFTVID

	1616 … 1700
P. textilis vF5 (short)	KDCKLPMGLASGIIQDSQISASGHVGYWEPKLARLNNTAIFNAWSIIKKEHEHPWIQIDLQRQVVITGIQTQGTVQLLQHSYTVE
O. microlepidotus vF5 (short)	KDCKLPMGLASGIIQDSQISASGHVEYWEPKLARLNNTGMFNAWSIIKKEHEHPWIQIDLQRQVVITGIQTQGTVQLLKHSYTVE
P. textilis F5 (short)	KDCKLPMGLASGIIQDSQISASGHVGYWEPKLARLNNTGKYNAWSIIKKEHEHPWIQIDLQRQVVITGIQTQGAMQLLKHLYTVE
O. microlepidotus F5 (short)	KDCKLPMGLASGIIQDSQISASGHVGYWEPKLARLNNPGKYNAWSIIKKEHELPWIQIDLQRQVVITGIQTQGAMQLLKHLYTVE
P. textilis F5	KDCKLPMGLASGIIQDSQISASGHVGYWEPKLARLNNTGKYNAWSIIKKEHEHPWIQIDLQRQVVITGIQTQGAMQLLKHLYTVE
O. microlepidotus F5	KDCKLPMGLASGIIQDSQISASGHVGYWEPKLARLNNPGKYNAWSIIKKEHELPWIQIDLQRQVVITGIQTQGAMQLLKHLYTVE
N. scutatus F5	KDCKLPMGLASGIIQDSQISASGHVGYWEPKLARLNNPGKYNAWSIIKKEHEHPWIQIDLQRQVVITGIQTQGAMQLLKHLYTVE
B. candidus F5	KDCKLPMGLASGIIQDSQITASGHVGYWEPKLARLNNTGKYNAWSIIKREHEHPWIQVDLQRQVVITGIQTQGAMQLLKHLYTVE
N. helvetica F5	KDCKLPMGLESGIIQDSQIIASDHLGFWEPKLARLNNAGKYNAWSTIKKENEHPWIQVDLQREVVITGIQTQGAMQLVKHLYTVE

	1701 … 1785
P. textilis vF5 (short)	YFVTYSEDGQNWITFKGRHSETQMHFEGNSDGTTVKENHIDPPIIARYIRLHPTKFYNRPTFRIELLGCEVEGCSVPLGMESGAI
O. microlepidotus vF5 (short)	YFVTYSKDGQNWITFKGRHSETQMHFEGNSDGTTVKENHIDPPIIARYIRLHPTKFYNTPTFRIELLGCEVEGCSVPLGMESGAI
P. textilis F5 (short)	YFFTYSKDGQNWITFKGRHSETQMHFEGNSDGTTVKENHIDPPIIARYIRLHPTKFHNRPTFRIELLGCEVEGCSVPLGMESGAI
O. microlepidotus F5 (short)	YFVTYSKDGQNWITFKGRHSETQMHFEGNSDGTTVKENHTDPPIIARYIRLHPTKFHNRPTFRIELLGCEVEGCSVPLGMESGAI
P. textilis F5	YFFTYSKDGQNWITFKGRHSETQMHFEGNSDGTTVKENHIDPPIIARYIRLHPTKFHNRPTFRIELLGCEVEGCSVPLGMESGAI
O. microlepidotus F5	YFVTYSKDGQNWITFKGRHSETQMHFEGNSDGTTVKENHTDPPIIARYIRLHPTKFHNRPTFRIELLGCEVEGCSVPLGMESGAI
N. scutatus F5	YFVTYSKDGQNWITFKGRHSETQMHFEGNSDGSTVKENHIDPPIIARYIRLHPTKFHNRPTFRIELLGCEVEGCSVPLGMESGAI
B. candidus F5	YFVTYSKDGQKWITFKGRHSKTQMHFKGNSDGTKVKENHIDPPIIARYIRLHPTKFYNRPTFRIELLGCEVEGCSVPLGMESGAI
N. helvetica F5	YCVAYSKDGRKWITFKGRHPKTEMHFEGNSDGTKVKENHIDPPIIARYIRLHPTKFYNRPTFRIELLGCEVEGCSMPLGMESGAI

	1786 … 1870
P. textilis vF5 (short)	KNSEITASSYKKTWWSSWEPFLARLNLEGGTNAWQPEVNNKDQWLQIDLQHLTKITSIITQGATSMTTSMYVKTFSIHYTDDNST
O. microlepidotus vF5 (short)	KNSEITASSYKKTWWSSWEPFLARLNLEGGTNAWQPEVNNKDQWLQIDLQHLTKITSIITQGATSMTTAMYVKTFSIHYTDDNST
P. textilis F5 (short)	KNSEITASSYKKTWWSSWEPSLARLNLKGRTNAWQPKVNNKDQWLQIDLQHLTKITSIITQGATSMTTSMYVKTFSIHYTDDNST
O. microlepidotus F5 (short)	KDSEITASSYKKTWWSSWEPFLARLNLKGRTNAWQPKVNNKDQWLQIDLQHLTKITSIITQGATSMTTAMYVKTFSIHYTDDNST
P. textilis F5	KNSEITASSYKKTWWSSWEPSLARLNLKGRTNAWQPKVNNKDQWLQIDLQHLTKITSIITQGATSMTTSMYVKTFSIHYTDDNST
O. microlepidotus F5	KDSEITASSYKKTWWSSWEPFLARLNLKGRTNAWQPKVNNKDQWLQIDLQHLTKITSIITQGATSMTTAMYVKTFSIHYTDDNST
N. scutatus F5	KNSEITASSYKKTWWSSWEPSLARLNLKGRTNAWQPKVNNKDQWLQIDLQHLTKITSIITQGATSMSTAMYVKTFSIHYTDDNST
B. candidus F5	KNSEITASSYKKTWWSSWEPSLARLNLKGRTNAWQPKVNNKDQWLQIDLQHLTKITSIVTQGATSMSTPMYVKTFSIHYSDDNST
N. helvetica F5	KNSEITASSYKKTWWSSWEPSLARLNLKGRTNAWQPKVNNKDQWLQIDLKHLTKITSIITQGATSMSTEMYVKTFSIHYSDENST

	1871 … 1931
P. textilis vF5 (short)	WKPYLDVRTSMEKVFTGNINSDGHVKHFFKPPILSRFIRIIPKTWNQYIALRIELFGCEVF
O. microlepidotus vF5 (short)	WKPYLDVRTSMEKVFTGNINSDGHVKHFFKPPILSRFIRIIPKTWNQYIALRIELFGCEVF
P. textilis F5 (short)	WKPYLDVRTSMEKVFTGNINSDGHVKHFFKPPILSRFIRIIPKTWNQYIALRIELFGCEVF
O. microlepidotus F5 (short)	WKPYLDVRTSMEKVFTGNINTDGHVKHFFKPPILSRFIRIIPKTWNQYIALRIELFGCEVF
P. textilis F5	WKPYLDVRTSMEKVFTGNINSDGHVKHFFKPPILSRFIRIIPKTWNQYIALRIELFGCEVF
O. microlepidotus F5	WKPYLDVRTSMEKVFTGNINTDGHVKHFFKPPILSRFIRIIPKTWNQYIALRIELFGCEVF
N. scutatus F5	WKPYLDVCTSMEKVFTGNINTDGHVKHFFKPPILSRFIRIIPKTWNQYIALRIELFGCEVF
B. candidus F5	WKPYLDVCTSMEKVFTGNMNTDGHVKHFFIPPILSRFIRIIPKTWNQYIALRIELFGCDVF
N. helvetica F5	WKPYLDVCTSTEKVFTGNVNTDGHVKHFFKPPILSRFIRIIPKTWNRYIALRIELFGCDVF

Fig S8. The alternatively spliced venom factor V protein lacks a large portion of its B-domain region. Amino acid sequence alignment of hypothetical protein translations of *F5* genes**.** The canonical coagulation factor V isoforms are denoted as *vF5* or *F5*, whereas the shorter alternatively spliced isoforms are denoted as *vF5* short or *F5* short. Residues shown in grey correspond to the signal peptide, and residues shown in bold indicate the B-domain region.

O.microlepidotus vF5	MGRYSVSPVPKCLLLMFLGWSGLKYYQVNAAQLREYRIAAQLEDWDY**N**PQPEELSRLSESELTFKKIVYREYELDFKQEKPRDEL
O. microlepidotus vF5 (short)	MGRYSVSPVPKCLLLMFLGWSGLKYYQVNAAQLREYRIAAQLEDWDY**N**PQPEELSRLSESELTFKKIVYREYELDFKQEKPRDEL
O. microlepidotus F5	MGRYSVSPVPKCLLLMFLGWSGLKYYQVNAAQLREYRIAAQLEDWDY**S**PQPEELSRLSESELTFKKIVYREYELDFKQEKPRDEL
transcript/12353	MGRYSVSPVPKCLLLMFLGWSGLKYYQVNAAQLREYRIAAQLEDWDY**N**PQPEELSRLSESELTFKKIVYREYELDFKQEKPRDEL
O. microlepidotus F5 (short)	MGRYSVSPVPKCLLLMFLGWSGLKYYQVNAAQLREYRIAAQLEDWDY**S**PQPEELSRLSESELTFKKIVYREYELDFKQEKPRDEL
transcript/61854	MGRYSVSPVPKCLLLMFLGWSGLKYYQVNAAQLREYRIAAQLEDWDY**N**PQPEELSRLSESELTFKKIVYREYELDFKQEKPRDEL
O.microlepidotus vF5	SGLLGPTLRGEVGDILIIYFKNFATQPVSIHPQSAVYNKWSEGSSYSDGTSDVERLDDAVPPGQSFKYVWNITAEIGPKKADPPC
O. microlepidotus vF5 (short)	SGLLGPTLRGEVGDILIIYFKNFATQPVSIHPQSAVYNKWSEGSSYSDGTSDVERLDDAVPPGQSFKYVWNITAEIGPKKADPPC
O. microlepidotus F5	SGLLGPTLRGEVGDILIIYFKNFATQPVSIHPQSAVYNKWSEGSSYSDGTSDVERLDDAVPPGQSFKYVWNITAEIGPKKADPPC
transcript/12353	SGLLGPTLRGEVGDILIIYFKNFATQPVSIHPQSAVYNKWSEGSSYSDGTSDVERLDDAVPPGQSFKYVWNITAEIGPKKADPPC
O. microlepidotus F5 (short)	SGLLGPTLRGEVGDILIIYFKNFATQPVSIHPQSAVYNKWSEGSSYSDGTSDVERLDDAVPPGQSFKYVWNITAEIGPKKADPPC
transcript/61854	SGLLGPTLRGEVGDILIIYFKNFATQPVSIHPQSAVYNKWSEGSSYSDGTSDVERLDDAVPPGQSFKYVWNITAEIGPKKADPPC
O.microlepidotus vF5	LTY**A**YYSHVNMVRDFNSGLIGALLICKEGSLNANGAQKFFNREYVLMFSVFDESKNWYRKPSLQYTINGFANGTLPDVQACAYDH
O. microlepidotus vF5 (short)	LTY**A**YYSHVNMVRDFNSGLIGALLICKEGSLNANGAQKFFNREYVLMFSVFDESKNWYRKPSLQYTINGFANGTLPDVQACAYDH
O. microlepidotus F5	LTY**S**YYSHVNMVRDFNSGLIGALLICKEGSLNANGAQKFFNREYVLMFSVFDESKNWYRKPSLQYTINGFANGTLPDVQACAYDH
transcript/12353	LTY**A**YYSHVNMVRDFNSGLIGALLICKEGSLNANGAQKFFNREYVLMFSVFDESKNWYRKPSLQYTINGFANGTLPDVQACAYDH
O. microlepidotus F5 (short)	LTY**S**YYSHVNMVRDFNSGLIGALLICKEGSLNANGAQKFFNREYVLMFSVFDESKNWYRKPSLQYTINGFANGTLPDVQACAYDH
transcript/61854	LTY**A**YYSHVNMVRDFNSGLIGALLICKEGSLNANGAQKFFNREYVLMFSVFDESKNWYRKPSLQYTINGFANGTLPDVQACAYDH
O.microlepidotus vF5	ISWHLIGMSSSPEIFSVHFNGQTLEQNHYKVSTINLVGGASVTANMSVSRTGKWLISSLVAKHLQAGMYGYLNIKDCG**H**P**N**TLTR
O. microlepidotus vF5 (short)	ISWHLIGMSSSPEIFSVHFNGQTLEQNHYKVSTINLVGGASVTANMSVSRTGKWLISSLVAKHLQAGMYGYLNIKDCG**H**P**N**TLTR
O. microlepidotus F5	ISWHLIGMSSSPEIFSVHFNGQTLEQNHYKVSTINLVGGASVTANMSVSRTGKWLISSLVAKHLQAGMYGYLNIKDCG**N**P**D**TLTR
transcript/12353	ISWHLIGMSSSPEIFSVHFNGQTLEQNHYKVSTINLVGGASVTANMSVSRTGKWLISSLVAKHLQAGMYGYLNIKDCG**N**P**D**TLTR
O. microlepidotus F5 (short)	ISWHLIGMSSSPEIFSVHFNGQTLEQNHYKVSTINLVGGASVTANMSVSRTGKWLISSLVAKHLQAGMYGYLNIKDCG**N**P**D**TLTR
transcript/61854	ISWHLIGMSSSPEIFSVHFNGQTLEQNHYKVSTINLVGGASVTANMSVSRTGKWLISSLVAKHLQAGMYGYLNIKDCG**N**P**D**TLTR
O.microlepidotus vF5	KLSFRELRRIMNWEYFIAAEEITWDYAPEIPSSVDRRYKAQYLDNFSNFIGKKYKKAVFRQY**E**DGNFTKPTYAIWPKERGILGPV
O. microlepidotus vF5 (short)	KLSFRELRRIMNWEYFIAAEEITWDYAPEIPSSVDRRYKAQYLDNFSNFIGKKYKKAVFRQY**E**DGNFTKPTYAIWPKERGILGPV
O. microlepidotus F5	KLSFRELRRIMNWEYFIAAEEITWDYAPEIPSSVDRRYKAQYLDNFSNFIGKKYKKAVFRQY**K**DGNFTKPTYAIWPKERGILGPV
transcript/12353	KLSFRELRRIMNWEYFIAAEEITWDYAPEIPSSVDRRYKAQYLDNFSNFIGKKYKKAVFRQY**K**DGNFTKPTYAIWPKERGILGPV
O. microlepidotus F5 (short)	KLSFRELRRIMNWEYFIAAEEITWDYAPEIPSSVDRRYKAQYLDNFSNFIGKKYKKAVFRQY**K**DGNFTKPTYAIWPKERGILGPV
transcript/61854	KLSFRELRRIMNWEYFIAAEEITWDYAPEIPSSVDRRYKAQYLDNFSNFIGKKYKKAVFRQY**K**DGNFTKPTYAIWPKERGILGPV
O.microlepidotus vF5	I**K**AKVRDT**VT**IVFKNLASRPYSIYVHGVSVSKDAEGAIYPSDPKENITHGKAVEPGQVYTYKWTVLDTDEPTVKDSECITKLYHS
O. microlepidotus vF5 (short)	I**K**AKVRDT**VT**IVFKNLASRPYSIYVHGVSVSKDAEGAIYPSDPKENITHGKAVEPGQVYTYKWTVLDTDEPTVKDSECITKLYHS
O. microlepidotus F5	I**R**AKVRDT**IS**IVFKNLASRPYSIYVHGVSVSKDAEGAIYPSDPKENITHGKAVEPGQVYTYKWTVLDTDEPTVKDSECITKLYHS
transcript/12353	I**R**AKVRDT**IS**IVFKNLASRPYSIYVHGVSVSKDAEGAIYPSDPKENITHGKAVEPGQVYTYKWTVLDTDEPTVKDSECITKLYHS
O. microlepidotus F5 (short)	I**R**AKVRDT**IS**IVFKNLASRPYSIYVHGVSVSKDAEGAIYPSDPKENITHGKAVEPGQVYTYKWTVLDTDEPTVKDSECITKLYHS
transcript/61854	I**R**AKVRDT**IS**IVFKNLASRPYSIYVHGVSVSKDAEGAIYPSDPKENITHGKAVEPGQVYTYKWTVLDTDEPTVKDSECITKLYHS
O.microlepidotus vF5	AVDMTRDIASGLIGPLLVCK**L**KALSVKGVQNKADVEQHAVFAVFDENKSWYLEDNIKKYCSNPSSVKKDDPKFYKSNVMYTLNGY
O. microlepidotus vF5 (short)	AVDMTRDIASGLIGPLLVCK**L**KALSVKGVQNKADVEQHAVFAVFDENKSWYLEDNIKKYCSNPSSVKKDDPKFYKSNVMYTLNGY
O. microlepidotus F5	AVDMTRDIASGLIGPLLVCK**R**KALSVKGVQNKADVEQHAVFAVFDENKSWYLEDNIKKYCSNPSSVKKDDPKFYKSNVMYTLNGY
transcript/12353	AVDMTRDIASGLIGPLLVCK**R**KALSVKGVQNKADVEQHAVFAVFDENKSWYLEDNIKKYCSNPSSVKKDDPKFYKSNVMYTLNGY
O. microlepidotus F5 (short)	AVDMTRDIASGLIGPLLVCK**R**KALSVKGVQNKADVEQHAVFAVFDENKSWYLEDNIKKYCSNPSSVKKDDPKFYKSNVMYTLNGY
transcript/61854	AVDMTRDIASGLIGPLLVCK**R**KALSVKGVQNKADVEQHAVFAVFDENKSWYLEDNIKKYCSNPSSVKKDDPKFYKSNVMYTLNGY
O.microlepidotus vF5	ASDRTEVLGFHQSEVV**Q**WHLTSVGTVDEIVPVHLSGHTFLSKGKHQDILNLFPMSGESATVTMDNLGTWLLSSWGSCEMSNGMRL
O. microlepidotus vF5 (short)	ASDRTEVLGFHQSEVV**Q**WHLTSVGTVDEIVPVHLSGHTFLSKGKHQDILNLFPMSGESATVTMDNLGTWLLSSWGSCEMSNGMRL
O. microlepidotus F5	ASDRTEVLGFHQSEVV**E**WHLTSVGTVDEIVPVHLSGHTFLSKGKHQDILNLFPMSGESATVTMDNLGTWLLSSWGSCEMSNGMRL
transcript/12353	ASDRTEVLGFHQSEVV**E**WHLTSVGTVDEIVPVHLSGHTFLSKGKHQDILNLFPMSGESATVTMDNLGTWLLSSWGSCEMSNGMRL
O. microlepidotus F5 (short)	ASDRTEVLGFHQSEVV**E**WHLTSVGTVDEIVPVHLSGHTFLSKGKHQDILNLFPMSGESATVTMDNLGTWLLSSWGSCEMSNGMRL
transcript/61854	ASDRTEVLGFHQSEVV**E**WHLTSVGTVDEIVPVHLSGHTFLSKGKHQDILNLFPMSGESATVTMDNLGTWLLSSWGSCEMSNGMRL
O.microlepidotus vF5	RFLDANYDDEDEGNEEEEEDDGDIFADIFSPPEVVKKKE**EV**PVNFV**P**DPESD**AL**AKELGLLDDEDN**P**E**Q**S**RS**EQTEDDEEQLMIA
O. microlepidotus vF5 (short)	RFLDANYDDEDEGNEEEEEDDGDIFADIFSPPEVVKKKE**EV**PVNFV**P**DPESD**AL**AKELGLLDDEDN**P**E**Q**S**RS**EQTEDDEEQLMIA
O. microlepidotus F5	RFLDANYDDEDEGNEEEEEDDGDIFADIFSPPEVVKKKE**KD**PVNFV**S**DPESD**KI**AKELGLLDDEDN**Q**E**E**S**HN**EQTEDDEEQLMIA
transcript/12353	RFLDANYDDEDEGNEEEEEDDGDIFADIFSPPEVVKKKE**KD**PVNFV**S**DPESD**KI**AKELGLLDDEDN**Q**E**E**S**HN**EQTEDDEEQLMIA
O. microlepidotus F5 (short)	RFLDANYDDEDEGNEEEEEDDGDIFADIFSPPEVVKKKE**KD**PVNFV**S**DPESD**KI**AKELGLLDDEDN**Q**E**E**S**HN**EQTEDDEEQLMIA
transcript/61854	RFLDANYDDEDEGNEEEEEDDGDIFADIFSPPEVVKKKE**KD**PVNFV**S**DPESD**KI**AKELGLLDDEDN**Q**E**E**S**HN**EQTEDDEEQLMIA
O.microlepidotus vF5	**S**MLGLRSFKGSVAEEEL**KH**TALALEEDAHASGELSLNTHLVLINSTDAMRGNEELGT**Q**NNSYVNASETIYANYTSSTGEILISNI
O. microlepidotus vF5 (short)	**S**MLGLRSFKGSVAEEEL**KH**TALALEEDAHAS--
O. microlepidotus F5	**T**MLGLRSFKGSVAEEEL**NL**TALALEEDAHASGELSLNTHLVLINSTDAMRGNEELGT**R**NNSYVNASETIYANYTSSTGEILISNI
transcript/12353	**T**MLGLRSFKGSVAEEEL**NL**TALALEEDAHASGELSLNTHLVLINSTDAMRGNEELGT**R**NNSYVNASETIYANYTSSTGEILISNI
O. microlepidotus F5 (short)	**T**MLGLRSFKGSVAEEEL**NL**TALALEEDAHAS--
transcript/61854	**T**MLGLRSFKGSVAEEEL**NL**TALALEEDAHAS--
O.microlepidotus vF5	TAMIGILL**S**KKSQNEENITQISDSYKQMLAAEYLKAGRGLQS**K**DIEDLMSPEIIPSLKEQEENTFEKDIWDKKGERQILTEATLH
O. microlepidotus vF5 (short)	---
O. microlepidotus F5	TAMIGILL**S**KKSQNEENITQISDSYKQMLAAEYLKAGRGLQS**Q**DIEDLMSPEIIPSLKEQEENTFEKDIWDKKGERQILTEATLH
transcript/12353	TAMIGILL**F**KKSQNEENITQISDSYKQMLAAEYLKAGRGLQS**Q**DIEDLMSPEIIPSLKEQEENTFEKDIWDKKGERQILTEATLH
O. microlepidotus F5 (short)	---
transcript/61854	---

O.microlepidotus vF5 ALKEMHALFFYVQQKRNLSAFNITSEDMKFLPSTENDSTIDNSKANYFS**YD**YDYDYDYKEEEATAAANVEFSKVIINTISDDKKT
O. microlepidotus vF5 (short) ---
O. microlepidotus F5 ALKEMHALFFYVQQKRNLSAFNITSEDMKFLPSTENDSTIDNSKANYFS--YDYDYDYKEEEATAAANVEFSKVIINTISDDKKT
transcript/12353 ALKEMHALFFYVQQKRNLSAFNITSEDMKFLPSTENDSTIDNSKANYFS--YDYDYDYKEEEATAAANVEFSKVIINTISDDKKT
O. microlepidotus F5 (short) ---
transcript/61854 ---

O.microlepidotus vF5 VENNRSEVNLVSVPSIMPQPENLTSDATLDNFVSTTISKTTK**P**WNSSSHQRQKCLPKNTDMEWKKENYDTLADIPNDVDNKFQNS
O. microlepidotus vF5 (short) ---
O. microlepidotus F5 VENNRSEVNLVSVPSIMPQPENLTSDATLDNFVSTTISKTTK**A**WNSSSHQRQKCLPKNTDMEWKKENYDTLADIPNDVDNKFQNS
transcript/12353 VENNRSEVNLVSVPSIMPQPENLTSDATLDNFVSTTISKTTK**A**WNSSSHQRQKCLPKNTDMEWKKENYDTLADIPNDVDNKFQNS
O. microlepidotus F5 (short) ---
transcript/61854 ---

O.microlepidotus vF5 SRNASFYQENAMFPGKRKEKTDNSNGQLELGQPGITNKHDSQKDENCTINISQSFIKIQ*KKKKPKVLTPRTSKHLELPRNLNNS
O. microlepidotus vF5 (short) ---
O. microlepidotus F5 SRNASFYQENAMFPGKRKEKTDNSNGQLELGQPGITNKHDSQKDENCTINISQSFIKIQ**R**KKKKPKVLTPRTSKHLELPRNLNNS
transcript/12353 SRNASFYQENAMFPGKRKEKTDNSNGQLELGQPGITNKHDSQKDENCTINISQSFIKIQ**R**KKKKPKVLTPRTSKHLELPRNLNNS
O. microlepidotus F5 (short) ---
transcript/61854 ---

O.microlepidotus vF5 QLSNKPNHTKLLNEAKLTRKQDK**L**IVTIGLPVEDGDYQEYEIDNTDNDKSSSGSFEYELMYYNNPYTTDPRIDSNSARN**S**DDIAG
O. microlepidotus vF5 (short) --DPRIDSNSARN**S**DDIAG
O. microlepidotus F5 QLSNKPNHTKLLNEAKLTRKQDK**F**IVTIGLPVEDGDYQEYEIDNTDNDKSSSGSFEYELMYYNNPYTTDPRIDSNSARN**P**DDIAG
transcript/12353 QLSNKPNHTKLLNEAKLTRKQDK**L**IVTIGLPVEDGDYQEYEIDNTDNDKSSSGSFEYELMYYNNPYTTDPRIDSNSARN**P**DDIAG
O. microlepidotus F5 (short) --DPRIDSNSARN**P**DDIAG
transcript/61854 --DPRIDSNSARN**P**DDIAG

O.microlepidotus vF5 RYLRTINR**R**NKRRYYIAAEEVLWDYSPIGKSQVRS**LP**AKTTFKKAIFRSYLDDTFQTPSTGGEYEKHLGILGPIIRAEVDDVIEV
O. microlepidotus vF5 (short) RYLRTINR**R**NKRRYYIAAEEVLWDYSPIGKSQVRS**LP**AKTTFKKAIFRSYLDDTFQTPSTGGEYEKHLGILGPIIRAEVDDVIEV
O. microlepidotus F5 RYLRTINR**G**NKRRYYIAAEEVLWDYSPIGKSQVRS**RA**AKTTFKKAIFRSYLDDTFQTPSTGGEYEKHLGILGPIIRAEVDDVIEV
transcript/12353 RYLRTINR**G**NKRRYYIAAEEVLWDYSPIGKSQVRS**RA**AKTTFKKAIFRSYLDDTFQTPSTGGEYEKHLGILGPIIRAEVDDVIEV
O. microlepidotus F5 (short) RYLRTINR**G**NKRRYYIAAEEVLWDYSPIGKSQVRS**RA**AKTTFKKAIFRSYLDDTFQTPSTGGEYEKHLGILGPIIRAEVDDVIEV
transcript/61854 RYLRTINR**G**NKRRYYIAAEEVLWDYSPIGKSQVRS**RA**AKTTFKKAIFRSYLDDTFQTPSTGGEYEKHLGILGPIIRAEVDDVIEV

O.microlepidotus vF5 QFRNLASRPYSLHAHGLLYEKSSEGRSYDD**N**SPELFKKDDAIMPNGTYTYVWQVPPRSGPTDNTEKCKSWAYYSGVNPEKDIHSG
O. microlepidotus vF5 (short) QFRNLASRPYSLHAHGLLYEKSSEGRSYDD**N**SPELFKKDDAIMPNGTYTYVWQVPPRSGPTDNTEKCKSWAYYSGVNPEKDIHSG
O. microlepidotus F5 QFRNLASRPYSLHAHGLLYEKSSEGRSYDD**K**SPELFKKDDAIMPNGTYTYVWQVPPRSGPTDNTEKCKSWAYYSGVNPEKDIHSG
transcript/12353 QFRNLASRPYSLHAHGLLYEKSSEGRSYDD**K**SPELFKKDDAIMPNGTYTYVWQVPPRSGPTDNTEKCKSWAYYSGVNPEKDIHSG
O. microlepidotus F5 (short) QFRNLASRPYSLHAHGLLYEKSSEGRSYDD**K**SPELFKKDDAIMPNGTYTYVWQVPPRSGPTDNTEKCKSWAYYSGVNPEKDIHSG
transcript/61854 QFRNLASRPYSLHAHGLLYEKSSEGRSYDD**K**SPELFKKDDAIMPNGTYTYVWQVPPRSGPTDNTEKCKSWAYYSGVNPEKDIHSG

O.microlepidotus vF5 LIGPILICQKGMIDKYNRTIDIREFVLFFMVFDEEKSWYFPKSDKST**CE**EKLIGVQS**H**HTFPAINGIPYQLQGLMMYKDENVHWH
O. microlepidotus vF5 (short) LIGPILICQKGMIDKYNRTIDIREFVLFFMVFDEEKSWYFPKSDKST**CE**EKLIGVQS**H**HTFPAINGIPYQLQGLMMYKDENVHWH
O. microlepidotus F5 LIGPILICQKGMIDKYNRTIDIREFVLFFMVFDEEKSWYFPKSDKST**RA**EKLIGVQS**R**HTFPAINGIPYQLQGLMMYKDENVHWH
transcript/12353 LIGPILICQKGMIDKYNRTIDIREFVLFFMVFDEEKSWYFPKSDKST**RA**EKLIGVQS**R**HTFPAINGIPYQLQGLMMYKDENVHWH
O. microlepidotus F5 (short) LIGPILICQKGMIDKYNRTIDIREFVLFFMVFDEEKSWYFPKSDKST**RA**EKLIGVQS**R**HTFPAINGIPYQLQGLMMYKDENVHWH
transcript/61854 LIGPILICQKGMIDKYNRTIDIREFVLFFMVFDEEKSWYFPKSDKST**RA**EKLIGVQS**R**HTFPAINGIPYQLQGLMMYKDENVHWH

O.microlepidotus vF5 LLNMGGPKDIHVVNFHGQTFTEEGREDNQLGVLPLLPGTF**A**SIKMKPSKIGTWLLETEVGENQERGMQALFTVIDKDCKLPMGLA
O. microlepidotus vF5 (short) LLNMGGPKDIHVVNFHGQTFTEEGREDNQLGVLPLLPGTF**A**SIKMKPSKIGTWLLETEVGENQERGMQALFTVIDKDCKLPMGLA
O. microlepidotus F5 LLNMGGPKDIHVVNFHGQTFTEEGREDNQLGVLPLLPGTF**T**SIKMKPSKIGTWLLETEVGENQERGMQALFTVIDKDCKLPMGLA
transcript/12353 LLNMGGPKDIHVVNFHGQTFTEEGREDNQLGVLPLLPGTF**T**SIKMKPSKIGTWLLETEVGENQERGMQALFTVIDKDCKLPMGLA
O. microlepidotus F5 (short) LLNMGGPKDIHVVNFHGQTFTEEGREDNQLGVLPLLPGTF**T**SIKMKPSKIGTWLLETEVGENQERGMQALFTVIDKDCKLPMGLA
transcript/61854 LLNMGGPKDIHVVNFHGQTFTEEGREDNQLGVLPLLPGTF**T**SIKMKPSKIGTWLLETEVGENQERGMQALFTVIDKDCKLPMGLA

O.microlepidotus vF5 SGIIQDSQISASGHV**E**YWEPKLARLNN**T**G**MF**NAWSIIKKEHE**H**PWIQIDLQRQVVITGIQTQG**TV**QLLKH**S**YTVEYFVTYSKDGQ
O. microlepidotus vF5 (short) SGIIQDSQISASGHV**E**YWEPKLARLNN**T**G**MF**NAWSIIKKEHE**H**PWIQIDLQRQVVITGIQTQG**TV**QLLKH**S**YTVEYFVTYSKDGQ
O. microlepidotus F5 SGIIQDSQISASGHV**G**YWEPKLARLNN**P**G**KY**NAWSIIKKEHE**L**PWIQIDLQRQVVITGIQTQG**AM**QLLKH**L**YTVEYFVTYSKDGQ
transcript/12353 SGIIQDSQISASGHV**G**YWEPKLARLNN**P**G**KY**NAWSIIKKEHE**H**PWIQIDLQRQVVITGIQTQG**AM**QLLKH**L**YTVEYFVTYSKDGQ
O. microlepidotus F5 (short) SGIIQDSQISASGHV**G**YWEPKLARLNN**P**G**KY**NAWSIIKKEHE**L**PWIQIDLQRQVVITGIQTQG**AM**QLLKH**L**YTVEYFVTYSKDGQ
transcript/61854 SGIIQDSQISASGHV**G**YWEPKLARLNN**P**G**KY**NAWSIIKKEHE**H**PWIQIDLQRQVVITGIQTQG**AM**QLLKH**L**YTVEYFVTYSKDGQ

O.microlepidotus vF5 NWITFKGRHSETQMHFEGNSDGTTVKENH**I**DPPIIARYIRLHPTKF**Y**N**T**PTFRIELLGCEVEGCSVPLGMESGAIKNSEITASSY
O. microlepidotus vF5 (short) NWITFKGRHSETQMHFEGNSDGTTVKENH**I**DPPIIARYIRLHPTKF**Y**N**T**PTFRIELLGCEVEGCSVPLGMESGAIKNSEITASSY
O. microlepidotus F5 NWITFKGRHSETQMHFEGNSDGTTVKENH**T**DPPIIARYIRLHPTKF**H**N**R**PTFRIELLGCEVEGCSVPLGMESGAIKDSEITASSY
transcript/12353 NWITFKGRHSETQMHFEGNSDGTTVKENH**T**DPPIIARYIRLHPTKF**H**N**R**PTFRIELLGCEVEGCSVPLGMESGAIKDSEITASSY
O. microlepidotus F5 (short) NWITFKGRHSETQMHFEGNSDGTTVKENH**T**DPPIIARYIRLHPTKF**H**N**R**PTFRIELLGCEVEGCSVPLGMESGAIKDSEITASSY
transcript/61854 NWITFKGRHSETQMHFEGNSDGTTVKENH**I**DPPIIARYIRLHPTKF**H**N**R**PTFRIELLGCEVEGCSVPLGMESGAIKDSEITASSY

O.microlepidotus vF5 KKTWWSSWEPFLARLNL**E**G**G**TNAWQP**E**VNNKDQWLQIDLQHLTKITSIITQGATSMTTAMYVKTFSIHYTDDNSTWKPYLDVRTS
O. microlepidotus vF5 (short) KKTWWSSWEPFLARLNL**E**G**G**TNAWQP**E**VNNKDQWLQIDLQHLTKITSIITQGATSMTTAMYVKTFSIHYTDDNSTWKPYLDVRTS
O. microlepidotus F5 KKTWWSSWEPFLARLNL**K**G**R**TNAWQP**K**VNNKDQWLQIDLQHLTKITSIITQGATSMTTAMYVKTFSIHYTDDNSTWKPYLDVRTS
transcript/12353 KKTWWSSWEPFLARLNL**K**G**R**TNAWQP**K**VNNKDQWLQIDLQHLTKITSIITQGATSMTTAMYVKTFSIHYTDDNSTWKPYLDVRTS
O. microlepidotus F5 (short) KKTWWSSWEPFLARLNL**K**G**R**TNAWQP**K**VNNKDQWLQIDLQHLTKITSIITQGATSMTTAMYVKTFSIHYTDDNSTWKPYLDVRTS
transcript/61854 KKTWWSSWEPFLARLNL**K**G**R**TNAWQP**K**VNNKDQWLQIDLQHLTKITSIITQGATSMTTAMYVKTFSIHYTDDNSTWKPYLDVRTS

O.microlepidotus vF5	MEKVFTGNIN**S**DGHVKHFFKPPILSRFIRIIPKTWNQYIALRIELFGCEVF
O. microlepidotus vF5 (short)	MEKVFTGNIN**S**DGHVKHFFKPPILSRFIRIIPKTWNQYIALRIELFGCEVF
O. microlepidotus F5	MEKVFTGNIN**T**DGHVKHFFKPPILSRFIRIIPKTWNQYIALRIELFGCEVF
transcript/12353	MEKVFTGNIN**T**DGHVKHFFKPPILSRFIRIIPKTWNQYIALRIELFGCEVF
O. microlepidotus F5 (short)	MEKVFTGNIN**T**DGHVKHFFKPPILSRFIRIIPKTWNQYIALRIELFGCEVF
transcript/61854	MEKVFTGNIN**T**DGHVKHFFKPPILSRFIRIIPKTWNQYIALRIELFGCEVF

Fig S9. Full-length *F5* transcripts are present in inland taipan (*Oxyuranus microlepidotus*) venom gland tissue. Amino acid alignment of the hypothetical protein isoform translations encoded by the *F5* and *vF5* paralogs of *O. microlepidotus*, together with representative full-length translated Iso-seq reads of both isoforms from venom gland tissue corresponding to each *F5* paralog. The alignment provides qualitative evidence that both *F5* alternative splice variants are expressed in venom gland tissue. A single-base-pair indel in transcript/12353 that was absent in all other transcripts and therefore likely represents a technical artifact, was manually corrected to restore the reading frame. Amino acid residues that differ between the *F5* and vF5 genes are shown in bold.

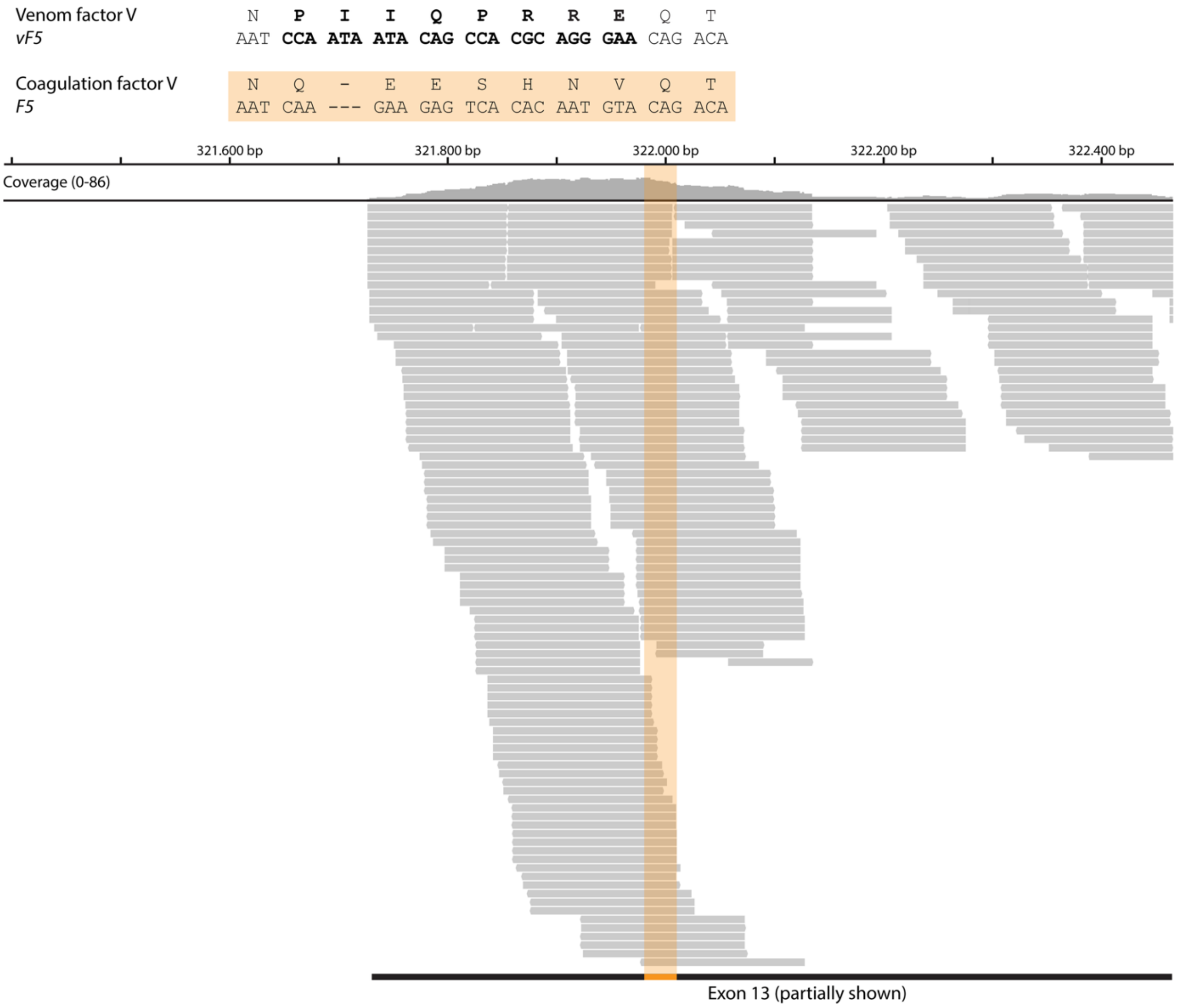

Fig S10. The hemostatic *F5* gene is expressed in Eastern brown snake (*Pseudonaja textilis*) venom gland tissue. One distinguishing feature between the *F5* and *vF5* paralogs in *P. textilis* is a unique structural variant in exon 13 of the *vF5* gene. The unique protein sequence and underlying codons are shown in bold for the *vF5* gene, with the corresponding protein sequence and codons in the *F5* gene highlighted in orange. We identified multiple short-read RNA-seq reads from *P. textilis* venom gland tissue that map to/cover this *F5*-specific region (highlighted in orange), providing additional evidence that the hemostatic *F5* gene is also transcribed in the venom gland of *P. textilis*.

Pseudonaja textilis vF5 MGRYSVSPVPKCLLLMFLGWSGLKYYQVNAAQLREYHIAAQLEDWDYNPQPEELSR**LSESDLTFKK**IVYR**EYELDFK**QEKPRDA
Pseudonaja textilis F5 (short) MGRYSVSPVPKCLLLMFLGWSGLKYYQVNAAQLREYRIAAQLEDWDYNPQPEELSR**LSESDLTFKK**IVYR**EYELDFK**QEKPRDA
Pseudonaja textilis F5 MGRYSVSPVPKCLLLMFLGWSGLKYYQVNAAQLREYRIAAQLEDWDYNPQPEELSR**LSESDLTFKK**IVYR**EYELDFK**QEKPRDA

Pseudonaja textilis vF5 LSGLLGPTLRGEVGDSLIIYFKNFATQPVSIHPQSAVYNK**WSEGSSYSDGTSDVERLDDAVPPGQSFK**YVWNITAEIGPKKADP
Pseudonaja textilis F5 (short) LSGLLGPTLRGEVGDILIIYFKNFATQPVSIHPQSAVYNK**WSEGSSYSDGTSDVERLDDAVPPGQSFK**YVWNITAEIGPKKADP
Pseudonaja textilis F5 LSGLLGPTLRGEVGDILIIYFKNFATQPVSIHPQSAVYNK**WSEGSSYSDGTSDVERLDDAVPPGQSFK**YVWNITAEIGPKKADP

Pseudonaja textilis vF5 PCLTYAYYSHVNMVRDFNSGLIGALLICK**EGSLNANGSQK**FFNREYVLMFSVFDESKNWYRKPSLQYTINGFANGTLPDVQACA
Pseudonaja textilis F5 (short) PCLTYAYYSHVNMVRDFNSGLIGALLICK**EGSLNANGAQK**FFNREYVLMFSVFDESKNWYRKPSLQYTINGFANGTLPDVQACA
Pseudonaja textilis F5 PCLTYAYYSHVNMVRDFNSGLIGALLICK**EGSLNANGAQK**FFNREYVLMFSVFDESKNWYRKPSLQYTINGFANGTLPDVQACA

Pseudonaja textilis vF5 YDHISWHLIGMSSSPEIFSVHFNGQTLEQNHYKVSTINLVGGASVTADMSVSRTGKWLISSLVAKHLQAGMYGYLNIK**DCGNPD**
Pseudonaja textilis F5 (short) YDHISWHLIGMSSSPEIFSVHFNGQTLEQNHYKVSTINLVGGASVTANMSVSRTGKWLISSLVAKHLQAGMYGYLNIK**DCGNPD**
Pseudonaja textilis F5 YDHISWHLIGMSSSPEIFSVHFNGQTLEQNHYKVSTINLVGGASVTANMSVSRTGKWLISSLVAKHLQAGMYGYLNIK**DCGNPD**

Pseudonaja textilis vF5 **TLTR**KLSFRELMKIKNWEYFIAAEEITWDYAPEIPSSVDRRYKAQYLDNFSNFIGKKYKKAVFRQYEDGNFTKPTYAIWPKER**G**
Pseudonaja textilis F5 (short) **TLTR**KLSFRELRRIMNWEYFIAAEEITWDYAPEIPSSVDRRYKAQYLDNFSNFIGKKYKKAVFR**QYKDSNFTK**PTYAIWPKERG
Pseudonaja textilis F5 **TLTR**KLSFRELRRIMNWEYFIAAEEITWDYAPEIPSSVDRRYKAQYLDNFSNFIGKKYKKAVFR**QYKDSNFTK**PTYAIWPKERG

Pseudonaja textilis vF5 **ILGPVIK**AK**VRDTVTIVFK**NLASRPYSIYVHGVSVSKDAEGAIYPSDPKENITHGK**AVEPGQVYTYK**WTVLDTDEPTVK**DSECI**
Pseudonaja textilis F5 (short) ILGPVIRAK**VRDTISIVFK**NLASRPYSIYVHGVSVSKDAEGAIYPSDPKENITHGK**AVEPGQVYTYK**WTVLDTDEPTVK**DSECI**
Pseudonaja textilis F5 ILGPVIRAK**VRDTISIVFK**NLASRPYSIYVHGVSVSKDAEGAIYPSDPKENITHGK**AVEPGQVYTYK**WTVLDTDEPTVK**DSECI**

Pseudonaja textilis vF5 **TKLYHSAVDMTR**DIASGLIGPLLVCKHKALSVKGVQNKADVEQHAVFAVFDENKSWYLEDNIK**KYCSNPSAVKKDDPKFYK**SNV
Pseudonaja textilis F5 (short) **TKLYHSAVDMTR**DIASGLIGPLLVCKHKALSVKGVQNKADVEQHAVFAVFDENKSWYLEDNIK**KYCSNPSTVKKDDPKFYK**SNV
Pseudonaja textilis F5 **TKLYHSAVDMTR**DIASGLIGPLLVCKHKALSVKGVQNKADVEQHAVFAVFDENKSWYLEDNIK**KYCSNPSTVKKDDPKFYK**SNV

Pseudonaja textilis vF5 MYTLNGYASDRTEVLRFHQSEVVQWHLTSVGTVDEIVPVHLSGHTFLSKGKHQDILNLFPMSGESATVTMDNLGTWLLSSWGSC
Pseudonaja textilis F5 (short) MYTLNGYASDRTEVLGFHQSEVVEWHLTSVGTVDEIVPVHLSGHTFLSKGKHQDILNLFPMSGESATVTMDNLGTWLLSSWGSC
Pseudonaja textilis F5 MYTLNGYASDRTEVLGFHQSEVVEWHLTSVGTVDEIVPVHLSGHTFLSKGKHQDILNLFPMSGESATVTMDNLGTWLLSSWGSC

Pseudonaja textilis vF5 EMSNGMRLRFLDANYDDEDEGNEEEEEDDGDIFADIFIPSEVVKKKEEVPVNFVPDPESDALAKELGLIDDEGNPIIQPR**REQT**
Pseudonaja textilis F5 (short) EMSNGMRLRFLDANYDDEDEGNEEEEEDDGDIFADIFIPPEVVKKKEKDPVNFVSDPESDKIAKELGLLDDEDNQ-EESHNVQT
Pseudonaja textilis F5 EMSNGMRLRFLDANYDDEDEGNEEEEEDDGDIFADIFIPPEVVKKKEKDPVNFVSDPESDKIAKELGLLDDEDNQ-EESHNVQT

Pseudonaja textilis vF5 **EDDEEQLMK**ASMLGLRSFKGSVAEEELKHTALALEEDAHAS---
Pseudonaja textilis F5 (short) EDDEEQLMIATMLGFRSFKGSVAEEELNLTALALEEDAHAS---
Pseudonaja textilis F5 EDDEEQLMIATMLGFRSFKGSVAEEELNLTALALEEDAHASGELSLNTHLVLINSTDAMRGNEELGTQNNSYVNASETIYANYT

Pseudonaja textilis vF5 --
Pseudonaja textilis F5 (short) --
Pseudonaja textilis F5 SSTGEILISNITAMIGILLSKRSQNEENITQISDSYKEMLAAEYLKAGRGLQSKDIEDLMSPEIIPSLKEQEENTFEKDIWDKK

Pseudonaja textilis vF5 --
Pseudonaja textilis F5 (short) --
Pseudonaja textilis F5 GERQILTEATLHALKEMHALFFYVQQKRNLSAFNITSEGMKFLPSTENDSTIDNSKANYFSYDYDYDYKEEEATAAANVEFSKV

Pseudonaja textilis vF5 --
Pseudonaja textilis F5 (short) --
Pseudonaja textilis F5 IINTISDDKKTVENNRSEVNLVSVPSIMPQPENLTSDATLDNFVSTTISKTTKTWNSSSHQRQKCLPKNTDMEWKKENYDTLAD

Pseudonaja textilis vF5 --
Pseudonaja textilis F5 (short) --
Pseudonaja textilis F5 IPNDVDNKFQNSSRNASFYQENAMFPGKRKEKTDNSNGQLELGQPGITNKHDSQKDENCTINISQSFIKIQRKKKKPKVLTPRT

Pseudonaja textilis vF5 ---DPR
Pseudonaja textilis F5 (short) ---DPR
Pseudonaja textilis F5 SKHLELPRNLNNSQLSNKPNHTKLLNEAKLTRKQDKLIVTIGLPVEDGDYQEYDIDNTDNDRSSSGSFEYELMYYNNPYTTDPR

Pseudonaja textilis vF5 **IDSNSARNPDDIAGR**YLRTINRGNKRRYYIAAEEVLWDYSPIGKSQVRSRAAKTTFKKAIFRSYLDDTFQTPSTGGEYEKHLGI
Pseudonaja textilis F5 (short) **IDSNSARNPDDIAGR**YLRTINRGNKRRYYIAAEEVLWDYSPIGKSQVRSRAAKTTFKKAIFRSYLDDTFQTPSTGGEYEKHLGI
Pseudonaja textilis F5 **IDSNSARNPDDIAGR**YLRTINRGNKRRYYIAAEEVLWDYSPIGKSQVRSRAAKTTFKKAIFRSYLDDTFQTPSTGGEYEKHLGI

Pseudonaja textilis vF5 LGPIIRAEVDDVIEIQFRNLASR**PYSLHAHGLLYEK**SSEGR**SYDDKSPELFKK**DDAIMPNGTYTYVWQVPPR**SGPTDNTEK**CKS
Pseudonaja textilis F5 (short) LGPIIRAEVDDVIEVQFRNLASR**PYSLHAHGLLYEK**SSEGR**SYDDKSPELFKK**DDAIMPNGTYTYVWQVPPR**SGPTDNTEK**CKS
Pseudonaja textilis F5 LGPIIRAEVDDVIEVQFRNLASR**PYSLHAHGLLYEK**SSEGR**SYDDKSPELFKK**DDAIMPNGTYTYVWQVPPR**SGPTDNTEK**CKS

Pseudonaja textilis vF5 WAYYSGVNPEKDIHSGLIGPILICQK**GMIDKYNR**TIDIREFVLFFMVFDEEKSWYFPKSDKSTCEEKLIGVQSLHTFPAINGIP
Pseudonaja textilis F5 (short) WAYYSGVNPEKDIHSGLIGPILICQK**GMIDKYNR**TIDIREFVLFFMVFDEEKSWYFPKSDKSTRAEKLIGVQSRHTFPAINGIP
Pseudonaja textilis F5 WAYYSGVNPEKDIHSGLIGPILICQK**GMIDKYNR**TIDIREFVLFFMVFDEEKSWYFPKSDKSTRAEKLIGVQSRHTFPAINGIP

Pseudonaja textilis vF5 YQLQGLTMYKDENVHWHLLNMGGPK**DIHVVNFHGQTFTEEGR**EDNQLGVLPLLPGTFASIKMKPSKIGTWLLETEVGENQERGM
Pseudonaja textilis F5 (short) YQLQGLTMYKDENVHWHLLNMGGPK**DIHVVNFHGQTFTEEGR**EDNQLGVLPLLPGTFASIKMKPSKIGTWLLETEVGENQERGM
Pseudonaja textilis F5 YQLQGLTMYKDENVHWHLLNMGGPK**DIHVVNFHGQTFTEEGR**EDNQLGVLPLLPGTFASIKMKPSKIGTWLLETEVGENQERGM

Pseudonaja textilis vF5	QALFTVIDKDCKLPMGLASGIIQDSQISASGHVGYWEPKLARLNNTAIFNAWSIIKKEHEHPWIQIDLQRQVVITGIQTQGTVQ
Pseudonaja textilis F5 (short)	QALFTVIDKDCKLPMGLASGIIQDSQISASGHVGYWEPKLARLNNTGKYNAWSIIKKEHEHPWIQIDLQRQVVITGIQTQGAMQ
Pseudonaja textilis F5	QALFTVIDKDCKLPMGLASGIIQDSQISASGHVGYWEPKLARLNNTGKYNAWSIIKKEHEHPWIQIDLQRQVVITGIQTQGAMQ
Pseudonaja textilis vF5	LLQHSYTVEYFVTYSEDGQNWITFK**GRHSETQMHFEGNSDGTTVKENHIDPPIIAR**YIRLHPTK**FYNRPTFR**IELLGCEVEGCS
Pseudonaja textilis F5 (short)	LLKHLYTVEYFFTYSKDGQNWITFK**GRHSETQMHFEGNSDGTTVKENHIDPPIIAR**YIRLHPTKFHNRPTFRIELLGCEVEGCS
Pseudonaja textilis F5	LLKHLYTVEYFFTYSKDGQNWITFK**GRHSETQMHFEGNSDGTTVKENHIDPPIIAR**YIRLHPTKFHNRPTFRIELLGCEVEGCS
Pseudonaja textilis vF5	VPLGMESGAIK**NSEITASSYKK**TWWSSWEPFLARLNLEGGTNAWQPEVNNKDQWLQIDLQHLTKITSIITQGATSMTTSMYVKT
Pseudonaja textilis F5 (short)	VPLGMESGAIK**NSEITASSYKK**TWWSSWEPSLARLNLKGRTNAWQPKVNNKDQWLQIDLQHLTKITSIITQGATSMTTSMYVKT
Pseudonaja textilis F5	VPLGMESGAIK**NSEITASSYKK**TWWSSWEPSLARLNLKGRTNAWQPKVNNKDQWLQIDLQHLTKITSIITQGATSMTTSMYVKT
Pseudonaja textilis vF5	FSIHYTDDNSTWKPYLDVRTSMEK**VFTGNINSDGHVKHFFKPPILSR**FIRIIPKTWNQYIALRIELFGCEVF
Pseudonaja textilis F5 (short)	FSIHYTDDNSTWKPYLDVRTSMEK**VFTGNINSDGHVKHFFKPPILSR**FIRIIPKTWNQYIALRIELFGCEVF
Pseudonaja textilis F5	FSIHYTDDNSTWKPYLDVRTSMEK**VFTGNINSDGHVKHFFKPPILSR**FIRIIPKTWNQYIALRIELFGCEVF

Fig. S11. Coagulation factor V protein is detected in Eastern brown snake (*Pseudonaja textilis*) venom. Amino acid sequence alignment of hypothetical F5 protein translations with tryptic peptides shown in bold. Red residues indicate peptides unique to the venom protein (vF5), purple residues indicate peptides unique to the hemostatic liver isoforms (F5 or F5 short), and black residues indicate peptides shared by both paralogs. Residues shown in grey correspond to the signal peptide. Tryptic peptides corresponding to the hemostatic form were detected in venoms from all individuals (N = 7).

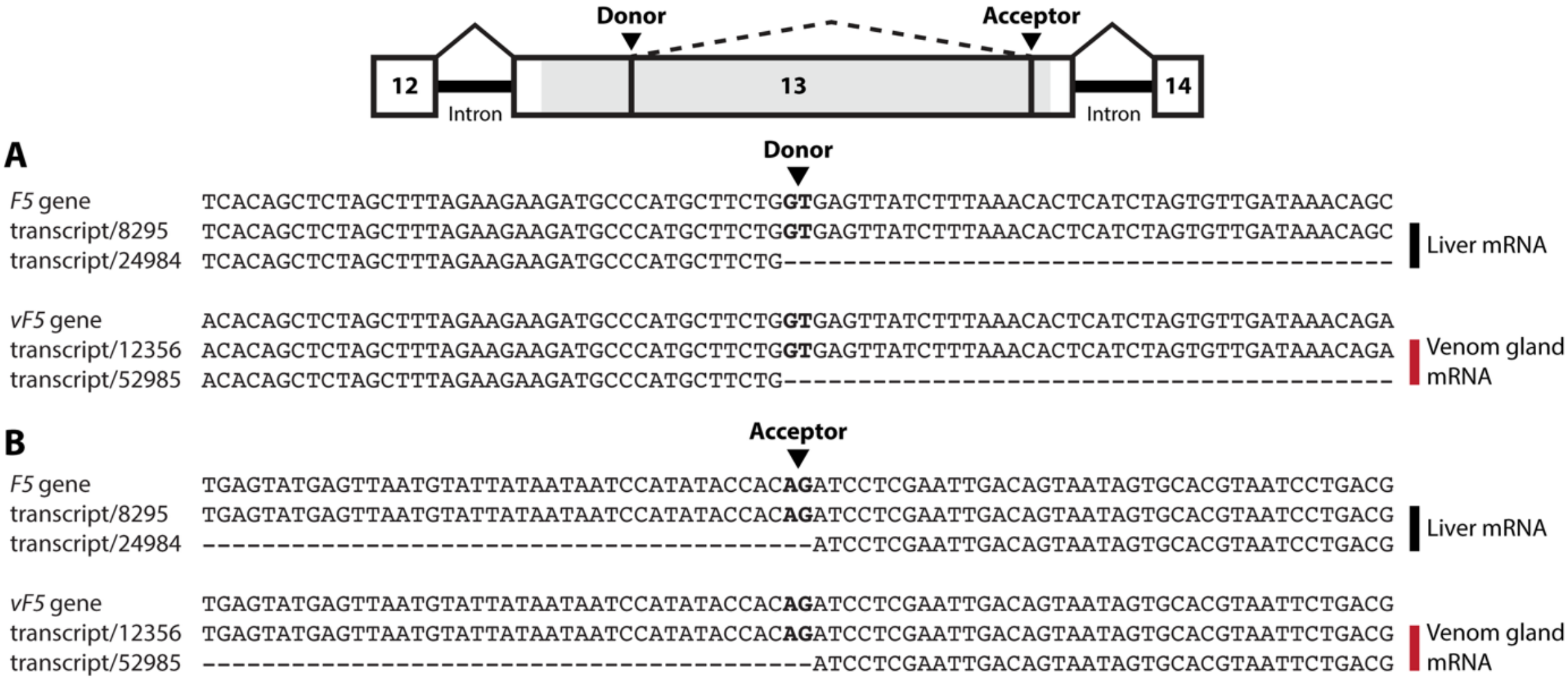

Fig. S12. *F5* gene paralogs are alternatively spliced within exon 13. DNA and mRNA sequence comparison at the donor (A) and acceptor (B) splice sites within exon 13 of both *F5* paralogs. The alignments include the *F5* and *vF5* genes as references with each of their corresponding full-length RNA transcripts expressed in liver or venom gland tissue of the inland taipan (*Oxyuranus microlepidotus*). The longer RNA isoform encodes the entire exon 13 (transcripts 8295 and 12356), whereas the shorter isoform (transcripts 24984 and 52985) retains only two short coding sequences at the 5′ and 3′ ends of exon 13. These segments are flanked by two canonical splice sites (donor GT and acceptor AG), indicating that the shorter isoform is generated through alternative splicing within exon 13.

Sequence	1–76
P. textilis Acidic PLA2 1	MHPAHLLVLLGVYVSLLGAARIPPLPLSLDDFSNLITCANRGSRSWLDYAHYGCFCGSGGSGT
P. textilis Acidic PLA2 2	MHPAHLLVLLGVCVSLLGAARIPPLPLSLVQFRILIKCANHNSRNVLDYADYGCYCGKGGSGT
O. microlepidotus Acidic PLA2 1 OS1	MHPAHLLVLLAVCVSLLGAARIPPLPLSLLDFANLIECANHGTRSALAYVHYGCYCGKGGRGT
N. scutatus Acidic PLA2 HTe	MYPAHLQVLLAVCVSLLGASSIPARPLNLYQFGNMIQCANHGRRPTRHYMDYGCYCGKGGSGT
P. textilis Acidic PLA2 textilotoxin D subunit	MHPAHLLVLLGVCVSRLGAASIPRPSLNIMLFGNMIQCTIPCEQSWLGYLDYGCYCGSGSSGI
O. microlepidotus Acidic PLA2 taipoxin γ subunit	MHPAHLLVLLAVCVSLLGSSEIPQPSLDFEQFSNMIQCTIPCGESCLAYMDYGCYCGPGGSGT
B. candidus Acidic PLA2 Bc-PL	MNPAHLLVLSALCVSLLGAANIPPQPLNLLQFNEMIECTIPGSFPLLDYMDYGCYCGTGGRGT
B. candidus Acidic PLA2 1	MNPAHFLVLAAVCVSLLGAANIPPQPLSFYRYTQMIQCTIRGSLTYLDYMDYGCYCGTGSRGT
B. candidus Acidic β bungarotoxin A3	MNPAHLLVLAAVCVSLLGAANIPPHPLNLINFMEMIRYTIPCEKTWGEYTNYGCYCGAGGSGR
B. candidus Basic β bungarotoxin A2	MNPAHLLVLSAVCVSLLGAANIPPHPFNLINFMKMIRYTIPCEKTWGEYVDYGCYCGVGGSGR
B. candidus Basic β bungarotoxin A1	MNPAHLLVLSAVCVSLLGAANIPPHPLNLINFMEMIRYTIPCEKTWGEYADYGCYCGAGGSGR
N. scutatus Basic PLA2 notechis 11'2	MYPAHLLVLLAVCVSLLGAASIPPQPLNLYQFGNMIQCANHGSRATWHYMDYGCYCGAGGSGT
N. scutatus Basic PLA2 notexin	MYPAHLLVLLAVCVSLLGAASIPPQPLNLVQFSYLIQCANHGSRATWHYMDYGCYCGAGGSGT
N. scutatus PLA2 1	MYPAHLLVLLAVCVSLLGATSIPPQPLNLYQFKNMIPCANHGRRPTWHYMDYGCYCSSGGSGT
N. scutatus Acidic PLA2	MYPAHLLVLLAVCVSLLGAASIPPLPLNVAQFDNMIECANYGSRPSWHYMEYGCYCGKEGSGT
P. textilis Basic PLA2 textilotoxin A subunit	MHPAHLLVLLGVCVSLLGASDIPPLPLNLVQFSYLIRCANKYKRPGWHYANYGCYCGSGGRGT
P. textilis Basic PLA2 textilotoxin B subunit	MHPAHLLVLLAVYVSLLGAARIPPLPLDLVEFGFMIRCANRNSQPAWQYMDYGCYCGKRGSGT
O. microlepidotus Neutral PLA2 taipoxin β subunit	MHPAHLLVLLAVCVSLLGASDIPPLPLNLLQFGFMIECAIRNRQPALDFMNYGCYCGTVGRGT
P. textilis Acidic PLA2 textilotoxin C subunit	MHPAHLLVLLGVYVSLLGAARIPPLPLNLIQFSNMIKCTIPGSQPLLDYANYGCYCGPGNNGT
O. microlepidotus Basic PLA2 taipoxin α subunit 1	MHPAHLLVLLAVCVSLLGASDIPPLPLNLLQFGFMIRCANRRSRPVWHYMDYGCYCGKGGRGT
O. microlepidotus Basic PLA2 taipoxin α subunit 2	MHPAHLLVLLAVCVSLLGASDIPPLPLNLAQFGFMIKCANGGSRPVSHYMDYGCYCGKGGSGT
O. microlepidotus Basic PLA2 taipoxin α subunit 3	MHPAHLLVLLAVCVSLLGASDIPPLPLNLAQFGFMIKCANGGSRPVSHYMDYGCYCGKGGSGT
O. microlepidotus Basic PLA2 taipoxin α subunit 4	MHPAHLLVLLAVCVSLLGASDIPPLPLNLAQFGFMIKCANGGSRPVSHYMDYGCYCGKGGSGT
O. microlepidotus Basic PLA2 taipoxin α subunit 5	MHPAHLLVLLAVCVSLLGASDIPPLPLNLAQFGFMIKCANGGSRPVSHYMDYGCYCGKGGSGT
O. microlepidotus Basic PLA2 taipoxin α subunit 6	MHPAHLLVLLAVCVSLLGASDIPPLPLNLAQFGFMIKCANGGSRPVSHYMDYGCYCGKGGSGT
P. textilis Group IB PLA2	MNPAHLLVLLAVCVSLLGASDIPPRPLNLIQFGNMIKCTIPGSRPLLDYADYGCYCGPGGSGT
O. microlepidotus Group IB PLA2	MNPAHLLVLLAVCVSLLGASDIPPLPLNLIQFGNMIKCTIPGSRPLLDYADYGCYCGPGGSGT
B. candidus Group IB PLA2	MNPAHLLVLSAVCVSLLGAANVAPQPRNLYQFRNMIKCTIPGSRPLLDYANYGCYCGKGGRGT
N. helvetica Group IB PLA2	MNLAHLLVPAALCVSLVAASALPSLPRNLLQFSNMIKCAIPGSRPVLDYADYGCYCGFGGSGT

Sequence	77–126
P. textilis Acidic PLA2 1	PVDDLDRCCQVHDNCFGDAEKLPACN**YLFSG**PYWNPYSYKCNEGE-ITCTDDNDECAAFICNC
P. textilis Acidic PLA2 2	PVDELDRCCQAHDYCYDDAEKLPACN**YRFSG**PYWNPYSYKCNEGE-VTCTDDNDECKAFICNC
O. microlepidotus Acidic PLA2 1 OS1	PVDDLDRCCQVHDDCYGEAEKLPACN**YLMSS**PYFNSYSYKCNEGK-VTCTDDNDECKAFICNC
N. scutatus Acidic PLA2 HTe	PVDELDRCCKTHDDCYGEAEKLPACN**YMMSG**PYYNTYSYECNEGE-LTCKDDNDECGAFICNC
P. textilis Acidic PLA2 textilotoxin D subunit	PVDDVDKCCKTHDECYYKAGQIPGCS**VQPNE**VFNVDYSYECNEGQ-LTCNESNNECEMAVCNC
O. microlepidotus Acidic PLA2 taipoxin γ subunit	PIDDLDRCCKTHDECYAEAGKLSACK**SVLSE**PNNDTYSYECNEGQ-LTCNDDNDECKAFICNC
B. candidus Acidic PLA2 Bc-PL	PVDMLDRCCKEHDDCYAQIKENPKCS**SLLNV**PYVKQYSFTCSEGD-LTCSDAAGTCARIVCDC
B. candidus Acidic PLA2 1	PVDELDRCCQTHDNCYAEAEEHPKCS**SLVKS**PYMNLYSYTCSGGT-ITCNADNDECGAFICNC
B. candidus Acidic β bungarotoxin A3	PIDALDRCCYVHDNCYGDAANIRDC-----**N**PKTQSYSYKLTKRT-IICYGAAGTCARVVCDC
B. candidus Basic β bungarotoxin A2	PIDALDRCCYVHDNCYGDAEKKHKC-----**N**PKMQSYSYKLTKRT-IICYGAAGTCARIVCDC
B. candidus Basic β bungarotoxin A1	PIDALDRCCYVHDNCYGDAEKKHKC-----**N**PKTQSYSYKLTKRT-IICYGAAGTCARIVCDC
N. scutatus Basic PLA2 notechis 11'2	PVDELDRCCKAHDDCYTEAGKK-GC-----**S**PKLTLYSWKCIEKT-PTC-NSKTGCERSVCDC
N. scutatus Basic PLA2 notexin	PVDELDRCCKIHDDCYGDAEKK-GC-----**S**PKMSAYDYYCGENG-PYCRNIKKKCLRFVCDC
N. scutatus PLA2 1	PVDELDRCCKTHDDCYTNIGTS-KC-----**N**PKSQIYSWKCGSDG-PTC-DSKTGCERSVCEC
N. scutatus Acidic PLA2	PVDELDRCCKAHDDCYTEAEKR-RC-----**H**PKFSAYSWKCGSDG-PTC-DSETGCKRTVCDC
P. textilis Basic PLA2 textilotoxin A subunit	PVDDVDRCCQAHDKCYEDAEKL-GC-----**Y**PKWTTYYYYCGANG-PYC-KTRTKCQRFVCNC
P. textilis Basic PLA2 textilotoxin B subunit	PVDDVDRCCQTHNECYDEAAKIPGC-----**K**PKWTFYFWQCGSGSQFTCRKSKDVCRNVVCDC
O. microlepidotus Neutral PLA2 taipoxin β subunit	PVDDLDRCCKTRNECYAEAEKH-GC-----**Y**PSLTTYRWQCGRVG-LHC-NSKTQCEVFVCAC
P. textilis Acidic PLA2 textilotoxin C subunit	PVDDVDRCCQAHDECYDEASNH-GC-----**Y**PELTLYDYYCDTGV-PYC-KARTQCQVFVCGC
O. microlepidotus Basic PLA2 taipoxin α subunit 1	PVDDLDRCCQVHDECYGEAERRFGC-----**S**PYWTLYSWKCYGTA-PSC-NTKTRCQRFVCRC
O. microlepidotus Basic PLA2 taipoxin α subunit 2	PVDELDRCCQVHDECYGEAEKRFKC-----**V**PYMTLYSWKCYGTA-PSC-NTKTDCQRFVCNC
O. microlepidotus Basic PLA2 taipoxin α subunit 3	PVDELDRCCQVHDECYGEAEKRFKC-----**V**PYMTLYSWKCYGTA-PSC-NTKTDCQRFVCNC
O. microlepidotus Basic PLA2 taipoxin α subunit 4	PVDELDRCCQVHDECYGEAEKRFKC-----**V**PFVTLYSWKCYGKA-PSC-NTKTDCQRFVCNC
O. microlepidotus Basic PLA2 taipoxin α subunit 5	PVDELDRCCQVHDECYGEAEKRFKC-----**V**PFVTLYSWKCYGKA-PSC-NTKTDCQRFVCNC
O. microlepidotus Basic PLA2 taipoxin α subunit 6	PVDELDRCCQVHDECYGEAEKRFKC-----**V**PYMTLYSWKCYGTA-PSC-NTKTDCQRFVCNC
P. textilis Group IB PLA2	PVDELDRCCQTHDNCYSQAKKHPACK**SLLDS**PYTKIYSYTCSGGN-LTCKDDNDECGAFICNC
O. microlepidotus Group IB PLA2	PVDELDRCCQTHDNCYSQAKKHPACK**SLLDS**PYTKIYSYTCSGGN-LTCKDDNDECGAFICNC
B. candidus Group IB PLA2	PVDMLDRCCQTHDNCYDQAMKHPACK**SFLDS**PYIKTYSYTCSGGT-VTCKDVKDKCARFVCDC
N. helvetica Group IB PLA2	PVDQLDRCCQTHDNCYSQAKKHPACK**SLLDS**PYTKTYSYTCSGGN-ITCQDNNDECEAFVCNC

	127 155
P. textilis Acidic PLA2 1	DRTAAICFAGATYNDENFMVTIKKKNICQ
P. textilis Acidic PLA2 2	DRTAAICFAGAPYNDENFMITTKKKNICQ
O. microlepidotus Acidic PLA2 1 OS1	DRTAAICFAGATYNDENFMISKKRNDICQ
N. scutatus Acidic PLA2 HTe	DRTAAICFARAPYNDANWDINLIAR--CQ
P. textilis Acidic PLA2 textilotoxin D subunit	DRAAAICFARFPYNKNYWSINTEIH--CR
O. microlepidotus Acidic PLA2 taipoxin γ subunit	DRTAVTCFAGAPYNDLNYNIGMIEH--CK
B. candidus Acidic PLA2 Bc-PL	DRTAALCFAEAPYKRRNFKIDYKTR--CQ
B. candidus Acidic PLA2 1	DRTAALCFAKAPYNEENKEIDISKR--CQ
B. candidus Acidic β bungarotoxin A3	DRTAALCFGDSEYIEGHKNIDTARF--CQ
B. candidus Basic β bungarotoxin A2	DRTAALCFGNSEYIERHKNIDTKRY--CR
B. candidus Basic β bungarotoxin A1	DRTAALCFGDSEYIERHKNIDTKRH--CQ
N. scutatus Basic PLA2 notechis 11'2	DAAAAKCFAKAPYNKKNYNINTKKR--CQ
N. scutatus Basic PLA2 notexin	DVEAAFCFAKAPYNNANWNIDTKKR--CQ
N. scutatus PLA2 1	DAAAAKCFAKAPYNDANWNIDTEKH--CQ
N. scutatus Acidic PLA2	DATAAKCFAKAPFNQANWNIDTETH--CQ
P. textilis Basic PLA2 textilotoxin A subunit	DVVAADCFASYPYNRRYWFYSNKKR--CR
P. textilis Basic PLA2 textilotoxin B subunit	DFKAALCLTRARYNSANYNIDIKTH--CR
O. microlepidotus Neutral PLA2 taipoxin β subunit	DLAAAKCLAQEDYNPAHFNINTKAR--CR
P. textilis Acidic PLA2 textilotoxin C subunit	DLAVAKCLAGATYNDENKNINTGER--CQ
O. microlepidotus Basic PLA2 taipoxin α subunit 1	DAKAAECFARSPYQSSNWNINTKAR--CR
O. microlepidotus Basic PLA2 taipoxin α subunit 2	DAKAAECFARSPYQNKNWNINTKAR--CK
O. microlepidotus Basic PLA2 taipoxin α subunit 3	DAKAAECFARSPYQNKNWNINTKAR--CK
O. microlepidotus Basic PLA2 taipoxin α subunit 4	DAKAAECFARSPYQNKNWNINTKAR--CK
O. microlepidotus Basic PLA2 taipoxin α subunit 5	DAKAAECFARSPYQNKNWNINTKAR--CK
O. microlepidotus Basic PLA2 taipoxin α subunit 6	DAKAAECFARSPYQNKNWNINTKAR--CK
P. textilis Group IB PLA2	DRTAAICFAGAPYNKENYNIDTTKH--CK
O. microlepidotus Group IB PLA2	DRTAAICFAGAPYNKENYNIDTTKH--CK
B. candidus Group IB PLA2	DRAAAMCFARAPYNKKYKKLDKSKY--CK
N. helvetica Group IB PLA2	DRSAAICFAGAPYNEEYKKLDTGKY--CK

Fig. S13. The pancreatic loop is intact in group IB but not in group IA phospholipase A_2 venom proteins. Amino acid sequence alignment of hypothetical protein translations of group I phospholipase A_2 (PLA_2) genes. Residues shown in grey encode the signal peptide and propeptide regions, and residues in bold indicate the pancreatic loop in group IB PLA_2, which is absent in group IA PLA_2 proteins because of a 5-amino-acid deletion. The procoagulant proteins are "*P. textilis* Acidic PLA2 1", "*P. textilis* Acidic PLA2 2" and putatively "*O. microlepidotus* Acidic PLA2 1 OS1" based on its orthologous relationship to "*P. textilis* Acidic PLA2 1".

	1 ... 85
P. textilis textilinin 2a	MSSGGLLLLLGLLTLWEVLTPVSSKDRPELCELPPDTGPCRVRFPSFYYNPDEQKCLEFIYGGCEGNANNFITKEECESTC--AA
P. textilis textilinin 2b	MSSGGLLLLLGLLTLWEVLTPVSSKDRPELCELPPDTGPCRVRFPSFYYNPDEQKCLEFIYGGCEGNANNFITKEECESTC--AA
P. textilis textilinin 3	MSSGGLLLLLGLLTLWEVLTPVSSKDRPNFCKLPAETGRCNAKIPRFYYNPRQHQCIEFLYGGCGGNANNFKTIKECESTC--AA
P. textilis textilinin 7	MSSGGLLLLLGLLTLWEVLTPVSSKDRPKFCELLPDTGSCEDFTGAFHYSTRDRECIEFIYGGCGGNANNFKTLEECESTC--AA
O. microlepidotus microlepidin 5	MSSGGLLLLLGLLTLWEVLTPVSSKDRPKFYELPADIGPCEDFTGAFHYSPREHECIEFIYGGCEGNANNFNTLEECET------
O. microlepidotus microlepidin 3	MSSGGLLLLLGLLTLWEVLTPVSSKDRPKFCELPADIGPCEDFTGAFHYSPREHECIEFIYGGCEGNANNFNTLEECESAC--AA
N. scutatus tigerin 3	MSSGGLLLLLGLLTLWEILTPVSSKDRPHFCHLPHDTGPCKRNIQAFYYNPVYHTCLKFIYGGCQGNSNNFKTIDECKRTC--AA
N. scutatus tigerin 4	MSSGGLLLLLGLLTLWAELTPVSSKDHPEFCELPADSGPCRGILRAFYYHPVHRTCQMFIYGGCYGNANNFKTIDECKRTC--AA
N. scutatus tigerin 1	MSSRGLLLLLGLLTLWAELTPVSSKDHPEFCELPADSGPCRGILHAFYYHPVHRTCLEFIYGGCYGNANNFKTIDECKRTC--AA
B. candidus KTSPI A	MSSGGLLLLLGLLTLCAELTPVSSKDRPKFCNVPPEPGRCNANVRAFYYNPRLRKCIEFTYGGCGGNANNFKSRGECKRTC--AE
B. candidus KTSPI C	MSSGGLLLLLGLLTLWTELTPVSSKNRPPFCNLLPEPGRCNAIVRAFYYNSRLRKCLEFPYGGCGGNANNFKTIDECQRTC--AG
B. candidus β-bungarotoxin B2a	MSSGGLLLLLGLLTLWAELTPVSSRKRHPDCDKPPDTKICQTVVRAFYYKPSAKRCVQFRYGGCNGNGNHFKSDHLCRCECLEYP
B. candidus β-bungarotoxin B2b	MSSGGLLLLLGLLTLWAELTPVSSRKRHPDCDKAPDTRICQTVVRAFYYKPSEKRCVQFRYGGCKGNGNHFKSDHLCRCECLEYR
B. candidus β-bungarotoxin B1a	MSSGGLLLLLGLLTLWAELTPVSSRKRHPDCDKPPDKGNCGSVRRAFYYDTKLKTCKPFPYRGCKGNGNHFKTETLCRCECLVYP
B. candidus β-bungarotoxin B1b	MSSGGLLLLLGLLTLCAELTPVSSRQRHRDCDKPPDKGNCGSVRRAFYYDTRLKTCKAFPYRGCNGNGNHFKTETLCRCECLVYP
B. candidus PILP1a	MSSGSLLLLLGLLTFWAQLTPVSTKDRP-NCDKAPDTERCKPNVHAFYYNPSARDCLQFVYGGCDGNGKHFRSKALCLFHC--HR
B. candidus PILP1b	MSSGGLLLLLGLLTLWAELTPVSSRQRH-HCDKAPDTGRCKPNVHAFYYNPSARDCLQFVYGGCDGNGKHFRSKALCLFHC--HR
B. candidus B5	MSSGGLLLLLGLLTLCAELTPVSSKDRHPYCNLPPDPGPCHDNKFAFYHHPASNKCKEFVYGGCGGNDNRFKTRNKCQCTC--SG

Fig. S14. Australian elapid Kunitz-type toxins possess key residues associated with Kv1 potassium channel binding. Amino acid sequence alignment of hypothetical protein translations of Kunitz-type toxin genes**.** Residues shown in grey encode the signal peptide, and residues associated with plasmin inhibition or specificity are highlighted in red (25), whereas residues associated with Kv1 potassium-channel binding are highlighted in blue (26). Note that the β-bungarotoxin paralogs are known potassium-channel inhibiting neurotoxins (27, 28).

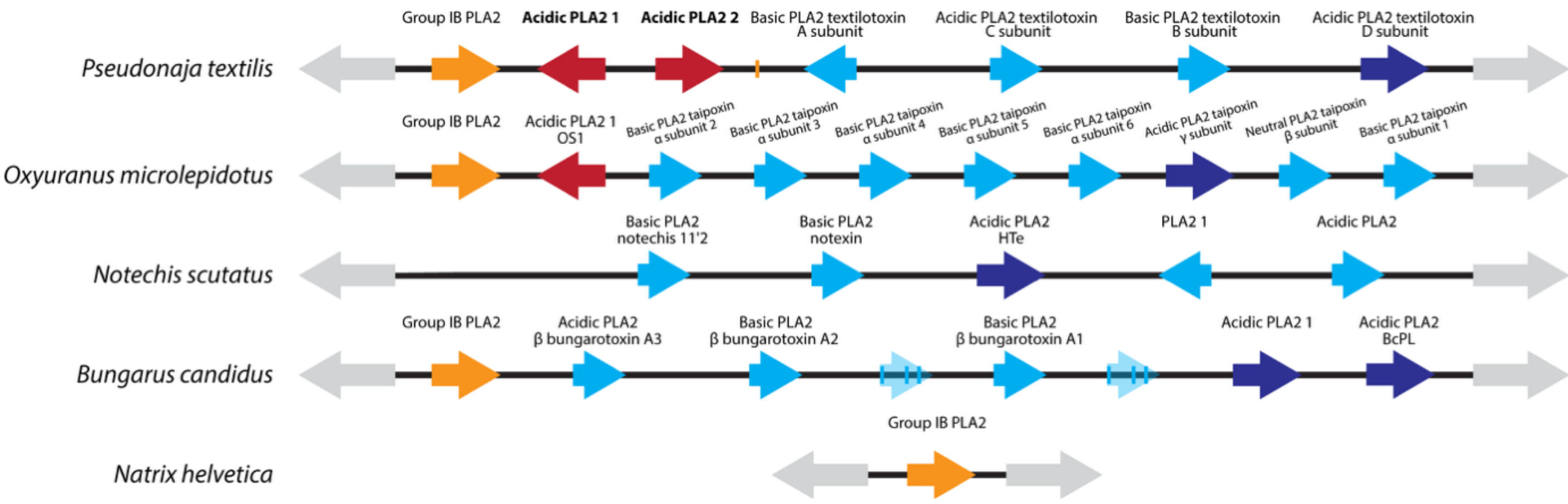

Fig. S15. Venom-expressed neurotoxic PLA2GIB proteins were neofunctionalized into procoagulant toxins. Protein identifications for the group I phospholipase genes shown in Fig. 5A, including procoagulant PLA2GIB proteins (red arrows), PLA2GIB proteins with neurotoxic or unknown activity (dark blue), neurotoxic PLA2GIA proteins (light blue), and the non-venom-expressed group IB PLA2 ortholog (orange). The *O. microlepidotus* Acidic PLA2 1 OS1 is a possible procoagulant protein based on its orthologous relationship with *P. textilis* Acidic PLA2 1; however, this protein has not been biochemically characterized.

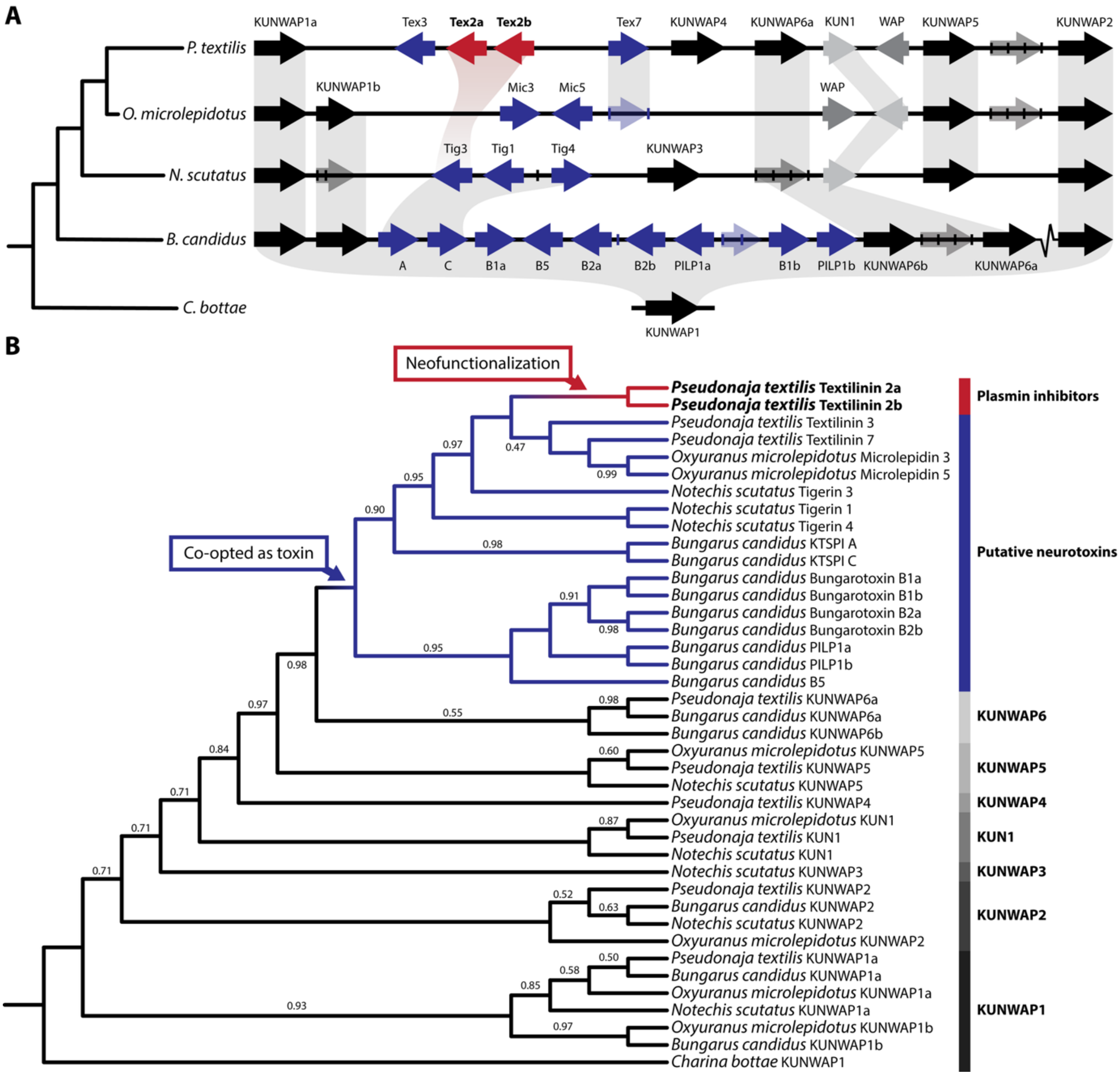

Fig. S16. Neofunctionalization of venom-expressed Kunitz-type toxins into plasmin-inhibiting proteins.

(A) Schematic showing the gene arrangement of venom-expressed and non-venom-expressed Kunitz-type serine protease inhibitor genes among different elapid snakes. The venom-expressed genes are either encoding plasmin-inhibiting toxins (red arrows) or putative neurotoxins (dark blue), or, waprins (dark grey). The non-venom-expressed encode either Kunitz proteins (light grey) or Kunitz–Waprin proteins (black). Pseudogenes are indicated by transparent arrows. The break-line symbol represents a genomic region with low confidence. The species phylogeny is based on reference (29).

(B) Protein phylogeny of Kunitz-type inhibitors reveals that the plasmin-inhibiting proteins (red branches) cluster within a clade of venom-expressed Kunitz-type toxins (blue branches), suggesting that the plasmin-inhibiting function evolved via neofunctionalization. This monophyletic clade of Kunitz-

type toxins clusters with a Kunitz–Waprin protein (KUNWAP6; black branches), suggesting that this ortholog represents the ancestral protein co-opted as a venom toxin. The phylogeny was rooted using the single-copy KUNWAP1 ortholog from *C. bottae* as the outgroup. Only Bayesian posterior probabilities less than 1.00 are shown.

Tables

Table S1. Comparison of gene expression levels between venom gland and liver tissues in *Pseudonaja textilis* using short-read RNA-sequencing.

Gene ID	Protein ID	Gene complex	Venom gland (TPM)	Liver (TPM)
NF2	Merlin	PLA2	69.59	34.15
PLA2G1B	Group IB PLA2	PLA2	3.99	0
PLA-1	Acidic PLA2 1	PLA2	140262.47	120.94
PLA-2	Acidic PLA2 2	PLA2	97315.99	99.91
PLA-3	Basic PLA2 textilotoxin A subunit	PLA2	23292.45	3
PLA-4	Acidic PLA2 textilotoxin C subunit	PLA2	42512.56	6
PLA-5	Basic PLA2 textilotoxin B subunit	PLA2	13785.71	2.87
PLA-6	Acidic PLA2 textilotoxin D subunit	PLA2	27505.92	0
THOC5	THO complex subunit 5	PLA2	394.56	335.28
KUNWAP1a	Kunitz–Waprin protein 1a	KUN	12.16	314.3
Textilinin-3	Textilinin 3	KUN	12137.17	0
*Textilinin-2a**	Textilinin 2a	KUN	274025.93	167.99
*Textilinin-2b**	Textilinin 2b	KUN	274025.93	167.99
Textilinin-7	Textilinin 7	KUN	49246.97	48
KUNWAP4	Kunitz–Waprin protein 4	KUN	23.94	0
KUNWAP6a	Kunitz–Waprin protein 6a	KUN	0	0
KUN1	Kunitz protein 1	KUN	0	0
WAP1	Waprin 1 (Textwaprin)	KUN	0	0
KUNWAP5	Kunitz–Waprin protein 5	KUN	0	0
KUNWAP2	Kunitz–Waprin protein 2	KUN	0	0

Abbreviation: TPM, Transcripts per Million. Similar TPM values were obtained using Kallisto (data not shown) (30). *Gene sequences of *textilinin-2a and textilinin-2b* are identical.

Table S2. Comparison of gene expression levels between venom gland and liver tissues in the Inland taipan (*Oxyuranus microlepidotus*) using short-read RNA-sequencing.

Gene ID	Protein ID	Gene complex	Venom gland (TPM)	Liver (TPM)
NF2	Merlin	PLA2	6.94	152.88
PLA2G1B	Group IB PLA2	PLA2	0.47	0
PLA-1	Acidic PLA2 1 OS1	PLA2	22561.82	0
*PLA-2**	Basic PLA2 taipoxin α subunit 2	PLA2	176034.55	0.61
*PLA-3**	Basic PLA2 taipoxin α subunit 3	PLA2	176034.55	0.61
*PLA-4***	Basic PLA2 taipoxin α subunit 4	PLA2	166106.5	0.3
*PLA-5***	Basic PLA2 taipoxin α subunit 5	PLA2	166106.5	0.3
PLA-6	Basic PLA2 taipoxin α subunit 6	PLA2	96369.47	0
PLA-7	Acidic PLA2 taipoxin γ subunit	PLA2	74955.84	0
PLA-8	Neutral PLA2 taipoxin β subunit	PLA2	53611.41	0
PLA-9	Basic PLA2 taipoxin α subunit 1	PLA2	42857.19	0
THOC5	THO complex subunit 5	PLA2	31.85	1273.86
KUNWAP1a	Kunitz–Waprin protein 1a	KUN	0	0
KUNWAP1b	Kunitz–Waprin protein 1b	KUN	0	0
Microlepidin-3	Microlepidin 3	KUN	880.78	0
Microlepidin-5	Microlepidin 5	KUN	218.67	0
WAP1	Waprin 1 (Omwaprin)	KUN	20991.74	0
KUN1	Kunitz protein 1	KUN	0.81	0
KUNWAP5	Kunitz–Waprin protein 5	KUN	1.98	0
KUNWAP2	Kunitz–Waprin protein 2	KUN	0	115.42

Abbreviation: TPM, Transcripts per Million. Similar TPM values were obtained using Kallisto (data not shown) (30). *Gene sequences of *PLA-2* and *PLA-3* are identical. **Gene sequences of *PLA-4* and *PLA-5* are identical.

SI References

1. S. Nurk *et al.*, HiCanu: accurate assembly of segmental duplications, satellites, and allelic variants from high-fidelity long reads. *Genome Research* **30**, 1291-1305.
2. H. Cheng, G. T. Concepcion, X. Feng, H. Zhang, H. Li, Haplotype-resolved de novo assembly using phased assembly graphs with hifiasm. *Nature Methods* **18**, 170-175 (2021).
3. G. Tans, J. W. Govers-Riemslag, J. L. van Rijn, J. Rosing, Purification and properties of a prothrombin activator from the venom of Notechis scutatus scutatus. *Journal of Biological Chemistry* **260**, 9366-9372 (1985).
4. V. S. Rao, J. S. Joseph, R. M. Kini, Group D prothrombin activators from snake venom are structural homologues of mammalian blood coagulation factor Xa. *Biochemical Journal* **369**, 635-642 (2003).
5. L. St. Pierre *et al.*, Comparative analysis of prothrombin activators from the venom of Australian elapids. *Molecular Biology and Evolution* **22**, 1853-1864 (2005).
6. S. Koren *et al.*, Canu: scalable and accurate long-read assembly via adaptive k-mer weighting and repeat separation. *Genome Research* 10.1101/gr.215087.116, gr.215087.215116 (2017).
7. I. H. Rossetto, A. J. Ludington, B. F. Simões, N. Van Cao, K. L. Sanders, Dynamic expansions and retinal expression of spectrally distinct short-wavelength opsin genes in sea snakes. *Genome Biology and Evolution* **16** (2024).
8. A. J. Ludington, J. M. Hammond, J. Breen, I. W. Deveson, K. L. Sanders, New chromosome-scale genomes provide insights into marine adaptations of sea snakes (Hydrophis: Elapidae). *BMC Biology* **21**, 284 (2023).
9. J. L. Grismer *et al.*, Reference genome of the rubber boa, Charina bottae (Serpentes: Boidae). *Journal of Heredity* **113**, 641-648 (2022).
10. S. F. Altschul *et al.*, Gapped BLAST and PSI-BLAST: a new generation of protein database search programs. *Nucleic Acids Research* **25**, 3389-3402 (1997).
11. A. Larsson, AliView: a fast and lightweight alignment viewer and editor for large datasets. *Bioinformatics* **30**, 3276-3278 (2014).
12. J. van Thiel *et al.*, A developmental shift in venom toxin expression underpins a major prey switch in Australian brown snakes. *BMC Biology* 10.1186/s12915-026-02699-0 (2026).

13. A. M. Bolger, M. Lohse, B. Usadel, Trimmomatic: a flexible trimmer for Illumina sequence data. *Bioinformatics* **30**, 2114-2120 (2014).

14. B. Li, C. N. Dewey, RSEM: accurate transcript quantification from RNA-Seq data with or without a reference genome. *BMC Bioinformatics* **12**, 323 (2011).

15. B. Langmead, S. L. Salzberg, Fast gapped-read alignment with Bowtie 2. *Nature Methods* **9**, 357-359 (2012).

16. H. Li, Aligning sequence reads, clone sequences and assembly contigs with BWA-MEM. *arXiv preprint arXiv:1303.3997* (2013).

17. P. Danecek *et al.*, Twelve years of SAMtools and BCFtools. *GigaScience* **10**, giab008 (2021).

18. J. T. Robinson *et al.*, Integrative genomics viewer. *Nature Biotechnology* **29**, 24-26 (2011).

19. H. Thorvaldsdóttir, J. T. Robinson, J. P. Mesirov, Integrative Genomics Viewer (IGV): high-performance genomics data visualization and exploration. *Briefings in Bioinformatics* **14**, 178-192 (2013).

20. A. T. Kong, F. V. Leprevost, D. M. Avtonomov, D. Mellacheruvu, A. I. Nesvizhskii, MSFragger: ultrafast and comprehensive peptide identification in mass spectrometry–based proteomics. *Nature Methods* **14**, 513-520 (2017).

21. F. Yu *et al.*, Identification of modified peptides using localization-aware open search. *Nature Communications* **11**, 4065 (2020).

22. R. C. Edgar, MUSCLE: multiple sequence alignment with high accuracy and high throughput. *Nucleic Acids Research* **32**, 1792-1797 (2004).

23. F. Ronquist *et al.*, MrBayes 3.2: Efficient Bayesian phylogenetic inference and model choice across a large model space. *Systematic Biology* **61**, 539-542 (2012).

24. M. A. Reza, T. N. Minh Le, S. Swarup, R. Manjunatha Kini, Molecular evolution caught in action: gene duplication and evolution of molecular isoforms of prothrombin activators in Pseudonaja textilis (brown snake). *Journal of Thrombosis and Haemostasis* **4**, 1346-1353 (2006).

25. E.-K. I. Millers *et al.*, The structure of human microplasmin in complex with textilinin-1, an aprotinin-like Inhibitor from the Australian brown snake. *PLOS ONE* **8**, e54104 (2013).

26. S. Gasparini *et al.*, Delineation of the functional site of alpha-dendrotoxin: The functional topographies of dendrotoxins are different but share a conserved core with those of other Kv1 potassium channel-blocking toxins. *Journal of Biological Chemistry* **273**, 25393-25403 (1998).

27. C. G. Benishin, Potassium channel blockade by the B subunit of beta-bungarotoxin. *Molecular Pharmacology* **38**, 164-169 (1990).

28. P.-F. Wu, S.-N. Wu, C.-C. Chang, L.-S. Chang, Cloning and functional expression of B chains of β-bungarotoxins from Bungarus multicinctus (Taiwan banded krait). *Biochemical Journal* **334**, 87-92 (1998).

29. H. Zaher *et al.*, Large-scale molecular phylogeny, morphology, divergence-time estimation, and the fossil record of advanced caenophidian snakes (Squamata: Serpentes). *PLOS ONE* **14**, e0216148 (2019).

30. N. L. Bray, H. Pimentel, P. Melsted, L. Pachter, Near-optimal probabilistic RNA-seq quantification. *Nature Biotechnology* **34**, 525-527 (2016).
